# The Connection Between Socioeconomic Factors and Dietary Habits of Children with Down Syndrome in Croatia

**DOI:** 10.3390/foods14111910

**Published:** 2025-05-28

**Authors:** Maja Ergović Ravančić, Valentina Obradović, Jadranka Vraneković

**Affiliations:** 1 Faculty of Tourism and Rural Development in Požega, Josip Juraj Strossmayer University of Osijek, Vukovarska 17, 34000 Požega, Croatia; mergovic@ftrr.hr; 2 Faculty of Medicine, University of Rijeka, B. Branchetta 20, 51000 Rijeka, Croatia; jadranka.vranekovic@medri.uniri.hr

**Keywords:** Down syndrome, diet, socioeconomic factors, nutrition, children

## Abstract

Children with Down syndrome often face significant feeding difficulties and health comorbidities that may contribute to undernutrition or obesity. This study assessed dietary habits and nutritional status among 104 children with Down syndrome in Croatia, representing 11.5% of this population. Results showed that over 30% of children aged 1 to 15 were overweight. Over 60% never consumed whole grain bread, while more than 50% avoided fish, nuts, or seeds. Despite rural families more frequently producing their own food (meat *p* = 0.009; fruits/vegetables *p* = 0.035), no significant improvement was observed in the children’s diets compared to their urban counterparts. Urban children consumed milk (*p* = 0.008) and fermented dairy (*p* = 0.005) more often. Children of university-educated mothers had higher vegetable (*p* = 0.031), meat (*p* = 0.025), olive oil (*p* = 0.003), and nut (*p* = 0.029) consumption, and a lower intake of processed meats (*p* = 0.008) and salty snacks (*p* = 0.040). Families spending less than 50% of income on food also showed significantly healthier dietary patterns. Feeding difficulties in children with Down syndrome are commonly associated with sensory sensitivities, oral-motor impairments, and comorbid medical conditions. These challenges are often intensified by parental anxiety, delayed introduction of diverse foods, and inadequate professional support. Collectively, these factors contribute to selective eating, poor nutrient intake, and disordered eating behaviors. This study underscores the need for individualized nutritional interventions that address the unique physiological and sensory requirements of both children and adults with Down syndrome. Effective strategies should extend beyond general dietary recommendations to include early exposure to a variety of food textures, specialized feeding support, and the management of coexisting health conditions. Family education and engagement play a crucial role in achieving positive nutritional outcomes. Empowering parents and caregivers—especially those in socioeconomically disadvantaged or rural communities—can facilitate the alignment of food accessibility with healthy dietary practices. The findings of this research offer valuable guidance for the development and implementation of national strategies aimed at enhancing the nutrition and long-term health of individuals with Down syndrome.

## 1. Introduction

Down syndrome is a genetic disorder caused by the presence of a partial or complete extra copy of chromosome 21 [1]. The global prevalence of Down syndrome is approximately 1 in 1000–1100 live births [2]. It is the most common genetic cause of intellectual disability, accounting for approximately 15% of individuals with intellectual disabilities. Trisomy 21 leads to the overexpression of more than 500 genes and their corresponding proteins, which are involved in regulating numerous neuronal processes, including transcription, translation, synaptic plasticity, and neuronal cell development [3].

Down syndrome is characterized by a range of phenotypic features, including a small chin, upward slanting eyes, hypotonia, a flat nasal bridge, a large gap between the first and second toes, unique fingerprint patterns, short fingers, a single palmar crease, and a protruding tongue due to a small oral cavity and macroglossia [4,5]. In addition to these features, individuals with Down syndrome often present with associated health conditions such as congenital heart defects, hearing and vision impairments, thyroid dysfunction, gastrointestinal issues, cognitive impairments [6,7,8], central nervous system anomalies [9,10], obstructive sleep apnea, and generalized muscle hypotonia [11].

Feeding difficulties are common in infants, children, and adolescents with Down syndrome, potentially resulting in protein-energy malnutrition and/or inadequate fluid intake. Older children often struggle with foods that are hard in texture, have mixed consistencies (e.g., cereal in milk), require more chewing (e.g., meat), or demand sensory tolerance (e.g., raw vegetables, unpeeled fruits, or minimally processed meats and fish) [12,13]. Neurodevelopmental disorders and developmental delays frequently contribute to oral motor dysfunction and swallowing difficulties, which in turn impact the ability of children to consume a variety of food textures [14,15,16,17]. Despite increasing recognition of feeding and swallowing challenges in individuals with Down syndrome across the lifespan, this issue and the interventions targeting it remain under-researched [18].

Feeding children with Down syndrome is further complicated by oral sensory processing issues, which affect their response to intraoral stimuli. This often results in extreme food selectivity and reduced acceptance of certain flavors [16]. Such sensory challenges, combined with anatomical and physiological differences and frequent dental or occlusal anomalies, increase the likelihood of feeding difficulties [19,20]. Food texture is often the most influential sensory characteristic affecting food preferences [14,21,22]. Early dietary intervention is essential to reduce or prevent aversions to specific textures, as exposure to a variety of textures at a young age may promote acceptance of more complex textures later in life [22].

Children and adults with Down syndrome experience health and metabolic challenges throughout their lives, which differ from the general population and are often underrecognized, preventing timely and appropriate interventions. One common issue is excessive body weight and obesity, particularly in adulthood [23]. Altered body composition characterized by shorter limbs, longer torso, and hypotonia contributes to this increased risk of obesity [24].

Previous research indicate that the prevalence of overweight and obesity among individuals with Down syndrome ranges from 23% to 70%, with a marked increase in weight gain often occurring after the age of two [12,25,26]. The high prevalence of disordered eating in individuals with intellectual disabilities, including those with Down syndrome, is largely attributed to a lack of understanding of proper nutrition, as well as a dependence on caregivers who may not be adequately informed about healthy dietary practices [27]. Research has shown that inappropriate mealtime behaviors can hinder weight management. Individuals with Down syndrome frequently exhibit food selectivity, engage in continuous eating in the presence of food regardless of hunger, swallow without sufficient chewing, and consume large quantities of food or calorie-dense beverages in short periods [12,28,29].

While general nutritional guidelines for health and improved quality of life in individuals with Down syndrome mirror those for the general population, they require additional monitoring due to genetic and physiological differences. Nutritional recommendations must consider comorbid conditions that influence dietary needs [30]. Effective nutritional strategies involve a comprehensive approach encompassing diet, physical activity, and educational interventions [31]. These efforts should focus on empowering families who spend the most time with the child to lead the process. Studies have shown that active family involvement plays a key role in achieving positive outcomes in weight management [32]. Research on nutritional challenges in children and adolescents with Down syndrome remains limited. Existing studies often have small sample sizes, wide age ranges, and insufficient descriptions of participants’ health statuses. Although general health challenges in individuals with Down syndrome are well documented, their links to nutritional issues are not yet thoroughly explored. A greater focus on dietary habits is crucial for improving health and quality of life. The distinct clinical features of Down syndrome have significant nutritional implications and must be systematically addressed. Therefore, clinical screening for feeding difficulties and comprehensive nutritional assessments are essential [33]. Mothers’ work hours are likely to affect their time allocation towards activities related to children’s diet, activity, and well-being [34]. Although maternal employment offers numerous benefits, research indicates an association between maternal employment and increased risk of childhood obesity, particularly when mothers work longer hours or are employed full-time. Elements of the family environment that support healthy eating, such as the availability of nutritious foods at home, regular family meals, and parental support for children’s healthy eating habits, may be compromised when mothers are employed outside the home. Studies have shown that employed mothers are more likely to purchase pre-prepared foods, including fast food and take-out, consume meals outside the home, and report a reduction in the frequency of shared family meals [35]. Additionally, a correlation was observed between household income and the availability of healthy food in families with young children [36].

Taking all of the above into account, it has been observed that, in the Republic of Croatia, there is a lack of research addressing the impact of various socioeconomic and health-related factors on the dietary habits of children with Down syndrome. In order to lay the groundwork for the development of effective national strategies to promote healthy eating habits, it is first necessary to identify whether certain subgroups within this already vulnerable population are particularly at risk of not receiving a nutritionally balanced diet. Therefore, this study aims to assess the dietary habits and nutritional status of children with Down syndrome in Croatia, representing a significant portion of this population. Specifically, we seek to identify the impact of socioeconomic factors, such as maternal education, family income, and place of residence (urban vs. rural), on dietary behaviors. Our hypothesis is that children with Down syndrome from socioeconomically disadvantaged backgrounds and rural areas exhibit poorer dietary patterns compared to their urban and higher socioeconomic status counterparts. Furthermore, we aim to emphasize the necessity of individualized nutritional interventions that address the unique physiological and sensory challenges faced by this population. By generating comprehensive, population-based data, this research intends to inform and support the development of effective national strategies to improve nutrition and long-term health outcomes for children with Down syndrome in Croatia.

## 2. Materials and Methods

The study was conducted during the first half of 2023 using an online questionnaire administered via Microsoft Forms (Microsoft Corporation, Redmond, WA, USA). The survey targeted parents and caregivers of children with Down syndrome, aged between 6 months and 19 years, residing in the Republic of Croatia. Respondents were reached through social media groups that provide support to families of people with Down syndrome. Participation in the study was anonymous and voluntary. All participants were fully informed about the voluntary nature of their involvement and were advised of their right to withdraw from the study at any time without consequence.

**Figure 1:**
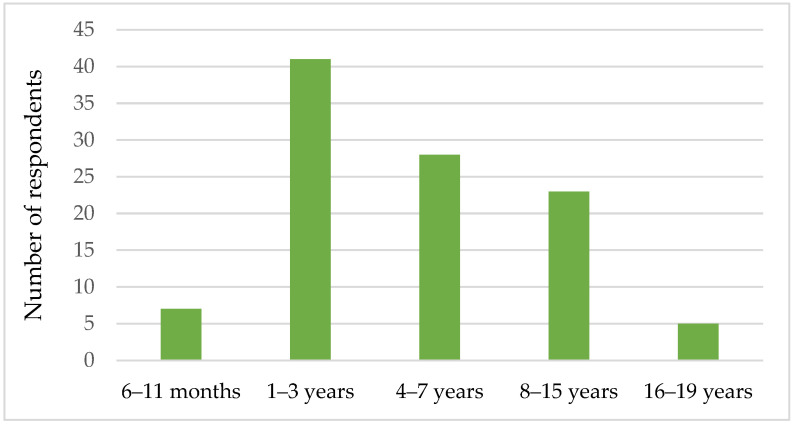
Age of the children with Down syndrome.

A total of 104 respondents, comprising parents and caregivers of children with Down syndrome aged between 6 months and 19 years, completed the survey, as presented in Figure 1. Of the children represented, 72 were male and 32 were female.

According to the most recent census conducted in 2021, the Republic of Croatia has a population of 3,871,833. The total number of individuals with Down syndrome, as reported by Croatian Institute for Public Health, is 2055, with 906 of them being children and adolescents up to 19 years of age [37]. Based on these figures, the present survey captured approximately 11.5% of the population of children with Down syndrome in the Republic of Croatia.

The initial section of the survey comprised questions regarding the child’s age, gender, place of residence, body weight, height, and overall health status. This was followed by items assessing the parents’ level of education, employment status, the monthly financial expenditure on food, as well as the family’s grocery shopping habits. In addition, information on the family’s place of residence (urban or rural) was collected, allowing for comparisons based on geographic location. These variables were included to explore the potential statistical significance of various family-related factors on the dietary habits of children with Down syndrome. This included the distinction between urban and rural areas, enabling further analysis of whether the child’s dietary habits varied according to their living environment.

In the third section, parents were asked to report the frequency with which their child consumed specific food items. A total of 28 food items, varying in origin and nutritional value, were included in this part of the survey.

### Data Analysis

Categorical data were summarized using absolute and relative frequencies. To assess differences in categorical variables between groups, the chi-square test was initially employed using Microsoft Excel (Microsoft Corporation, Redmond, WA, USA). However, due to the presence of small expected frequencies (less than 5 or, in some cases, less than 1), the application of the chi-square test was inappropriate in many instances. Consequently, we reported results of the chi-square test in the discussion section only where its use was justified and yielded statistically significant differences between groups. Given the limitations of the chi-square test, Fisher’s exact test was subsequently applied on a row-by-row basis using the “all others” comparison principle. In addition, Fisher’s exact test with Monte Carlo simulation with 10,000 permutations, was conducted at a 95% confidence level using R version 4.4.3 for Windows (R Foundation for Statistical Computing, Vienna, Austria). For Monte Carlo simulation, rows containing only zero values were excluded from the analysis prior to computation.

## 3. Results and Discussion

Given the limited availability of research and practical resources on the nutritional habits of children with Down syndrome in Croatia, the results in this study are presented in greater detail. This approach was chosen to ensure that the findings are accessible not only to researchers but also to parents, therapists, and other non-expert stakeholders who play a key role in managing the daily nutrition of these children. In many cases, families are left to navigate dietary challenges with insufficient professional support, which highlights the importance of providing clear and comprehensive data to inform real-life decisions and raise public awareness on this important but underrepresented issue.

### 3.1. Nutritional and Health Status of Children with Down Syndrome

A total of 25 of 104 children were born prematurely. The average birth weight was 2889.9 ± 821.3 g. When considering only full-term births, the average weight was 3146.64 ± 665.03 g, while the average weight of preterm infants was 2476 ± 693.43 g. The lowest recorded weight was 782 g, and the highest was 3750 g.

Table 1 presents respondents’ weight-for-age percentile, according to growth charts for children with Down syndrome [38]. Growth charts provide valuable insights by offering a general overview of the development of children with Down syndrome. However, these specific charts have certain limitations when compared to charts based on reference populations. These charts should be used as guidelines rather than definitive assessments, as the reference groups on which they are based typically consist of individuals with Down syndrome from specific geographic locations where the measurements were conducted [39,40].

A Swedish study revealed that European individuals with Down syndrome tend to be taller and have lower body mass index than their American counterparts, though high rates of overweight were found in both groups. While short stature is common, growth varies widely due to genetic, hereditary, and comorbid factors, highlighting the need for a holistic assessment of each child [41].

According to Table 1 the average percentile values across all age groups align with the recommended standards for the respective ages. However, the presented results indicate that some children are undernourished, specifically, girls up to the age of 3 and boys up to the age of 7. Nevertheless, a more significant health concern is the presence of overweight and obese children. Although none of the children aged 16 to 19 years had percentiles above the 85th, the small sample size within this age group limits the ability to draw definitive conclusions regarding the extent of the issue. Unfortunately, already after the first year of life, over 17% of girls and 16% of boys develop excess body weight. When the proportions of overweight and obese boys are combined for age groups ranging from 1 to 15 years, the figures reach approximately 30% or more. This highlights the critical importance of proper nutrition for children with Down syndrome.

The age range of the children included in the study was from 1 to 19 years. Health-related data, including diagnoses and ongoing treatments, were obtained through parent-reported information based on previously issued medical reports and official diagnoses by healthcare professionals.

According to the results of our research, 39 children are undergoing continuous therapy for a period longer than 3 months, with 26 reporting the use of synthetic thyroid hormone treatments for thyroid disorders, followed by medications for alleviating allergy symptoms, antiepileptic drugs, and medications for managing mental health disorders in individual cases. Among the prematurely born children (n = 25), only three reported no congenital health issues, while the remaining participants indicated the presence of various medical conditions, including congenital heart defects (atrial septal defect (ASD), ventricular septal defect (VSD), congenital anomalies, atrioventricular canal, tetralogy of Fallot, patent ductus arteriosus (PDA)) (n = 11), visual impairments (n = 6), brain injuries (n = 5), hypothyroidism/hyperthyroidism (n = 5), as well as other conditions such as leukemia, leukopenia, congenital gastrointestinal anomalies, hearing impairments, and psychiatric disorders. In the group of full-term children (n = 79), only twelve were reported to have no health problems. The rest presented with conditions such as congenital heart defects (n = 31), hypothyroidism/hyperthyroidism (n = 18), visual impairments (n = 19), hearing impairments (n = 14), and, in several cases, gastrointestinal anomalies and brain injuries. Chronic constipation was reported in both groups (n = 12), underscoring the importance of careful dietary management in daily nutrition.

Infants with Down syndrome can generally be breastfed successfully. However, a certain proportion of children may experience feeding difficulties due to the syndrome itself. These challenges may be related to anatomical differences in the digestive system, underdeveloped oral-motor skills, and the presence of hypotonia. Previous studies have shown that 84.6% of mothers cited their child’s sucking difficulties as the reason for not initiating or for discontinuing breastfeeding [16,42,43,44,45]. In addition to physical challenges, emotional factors have also been found to influence the decision to stop breastfeeding. Receiving a diagnosis of Down syndrome can be emotionally overwhelming for parents and may lead to stress, shock, and sadness, all of which can impact breastfeeding outcomes [46]. In our study, 50 children with Down syndrome were breastfed, while 54 were not.

The development of independent feeding skills in children with Down syndrome tends to progress more slowly than in typically developing children. However, the age at which solid foods are introduced is generally similar. A study [47] showed that 46% of children with Down syndrome were introduced to solid foods between 6 and 9 months of age, while 38% began between 9 and 12 months.

Feeding difficulties during the transition to solid foods often continue from early infancy and are primarily related to oral-motor challenges. These include a prolonged sucking reflex, uncoordinated or irregular chewing patterns, and poor bolus control [5,33]. Feeding problems are estimated to occur in approximately 50% to 80% of children with Down syndrome, compared to about 25% of typically developing children. For this reason, children with Down syndrome are often introduced to complementary foods later than typically developing children and World Health Organization recommendations [2]. This delay is attributed to factors such as medical complications, parental anxiety, and developmental delays. Parents of children with Down syndrome have been found to have more cautious and controlled feeding practices compared to those of typically developing children. This includes increased concerns about choking risks and the child’s weight, potentially leading to restrictive feeding behaviors [48].

According to our research, 8 out of 25 preterm infants experience feeding difficulties, primarily characterized by refusal to chew and eat solid food particles. Among the 79 full-term children, 19 also exhibit feeding problems, most commonly in the form of refusal to chew solid foods, as well as rejection of specific types of food, most often meat, fruit, or vegetables, or the rejection of certain food textures, such as crunchy or dry foods.

### 3.2. Analysis of Respondents’ Grocery Shopping Habits in Relation to Place of Residence

According to the definition provided by the Ministry of Justice of the Republic of Croatia, a city is a unit of local government that serves as the administrative center of a county and includes any settlement with more than 10,000 inhabitants, representing an urban, historical, natural, economic, and social entity [49]. Forty-five participants from this survey were from rural areas (less than 10,000 inhabitants), and 59 participants were from urban areas (more than 10,000 inhabitants).

The participants were asked to indicate where they most frequently purchase fresh meat, fruits and vegetables, and other food items. They were given the option to provide multiple responses (except in the case of other food items). The results are presented separately for individual responses as well as for responses that included multiple selections (Table 2).

As expected, there is a significant difference in shopping habits between urban and rural residents, primarily in the purchase of fresh meat. Statistically significant (*p* = 0.009) higher proportion of rural residents produce their own meat, while urban residents mostly purchase meat in retail chains (*p* = 0.034; *p* = 0.019 for single answers). Rural residents also tend to produce their own fruit and vegetables more than urban residents (*p* = 0.035). Regarding other food, urban residents buy it more often in large retail chains while rural residents buy it more often in small stores (*p* = 0.051).

Other studies have also confirmed significant differences in food purchasing behavior between rural and urban populations, influenced by factors such as the availability of stores, socioeconomic status, and accessibility of transportation [50]. The main findings highlighted that urban residents primarily shop at large grocery stores and supermarkets, benefiting from greater proximity and a wider variety of food options [51]. Rural shoppers often rely on small shops and buy food with a higher energy value than urban households [52]. In addition, home-based food production is more prevalent in rural areas; for example, in Brazil, 31% of rural households engage in home-based food production, compared to a much lower percentage in urban areas [53].

### 3.3. Dietary Habits of Children with Down Syndrome

#### 3.3.1. Analysis of Respondents’ Dietary Habits in Relation to Gender and Place of Residence

As previously stated, parents were asked to indicate how frequently their children with Down syndrome consume specific food items. A total of 28 different foods can be categorized into several groups: carbohydrate-rich foods, milk and dairy products, fruits and vegetables, protein-rich foods (meat/fish/eggs), oils and fats, nuts and seeds, unhealthy options such as sweets and snacks, and, finally, liquids. Table 3 presents the results according to the gender and place of residence of the participants.

A study conducted by Colić-Barić et al. [54] aimed to compare the nutrient intake and dietary behavior of urban and rural school children in Croatia. Using a quantified Food Frequency Questionnaire, data were collected from 315 urban and 163 rural children, with an average age of 12.5 and 12.6 years, respectively. The findings revealed that the consumption of fast food, soft drinks, and alcohol was more prevalent among urban children, but also that urban children had more adequate energy and nutrient intake.

Another study conducted in Croatia included 105 urban and 205 rural school-aged children. The results showed a higher prevalence of overweight and obesity among children in rural areas compared to children in urban areas. It was found that children from rural areas consume fewer fruits and vegetables and have a higher intake of sweet and fatty foods [55]. In a cross-sectional study’s data of 123,100 children aged 6–9 years across 19 countries participating in the World Health Organization European Childhood Obesity Surveillance Initiative (2015–2017), parents completed food-frequency questionnaires to assess children’s consumption patterns. The study found significant variability among countries regarding daily fruit and vegetable intake and soft drink consumption. In less than one-third of the countries, children attending rural schools had higher odds of not consuming fruits or vegetables daily and consuming soft drinks more than three times a week compared to their urban counterparts. The study emphasized the need for population-based interventions and policy strategies to improve access to healthy foods and promote healthy eating behaviors among children [56].

##### Carbohydrate-Rich Foods Consumption

When examining carbohydrate-rich foods, approximately half of both female and male with Down syndrome consume white bread five times a week or more frequently, i.e., on a daily basis (53.13% of female and 48.6% of male participants), while 28% of female and 38.89% of male participants report never consuming it. Conversely, 62.5% of both male and female participants report never consuming whole grain or brown bread. Participants from rural areas reported significantly more frequent consumption of white bread two or more times a day compared to those from urban areas (*p* = 0.043). Moreover, as many as 71% of rural participants stated that they never consume whole grain (black) bread. These findings suggest that white bread remains a staple in the diets of a substantial proportion of the sample. From a nutritional standpoint, this is concerning given that white bread is often low in fiber and essential micronutrients compared to whole grain alternatives. Whole grains are a valuable source of vitamin E and B vitamins, as well as minerals such as copper, selenium, zinc, iron, magnesium, and phosphorus, all of which are present in significant concentrations. Additionally, whole grains contain important phytochemicals, including phenolic acids and lignans, plant-based phytoestrogens that have been positively associated with maintaining cardiovascular health [57].

Our results showed that the majority of participants consume pasta, rice, or potatoes once or twice a week (generally between 70% and 80%), while whole grains are consumed five times a week or more by 46.88% of female and 43.05% of male participants. Children from urban areas tend to consume whole grain once a day or five times a week (44.07%) more often than children from rural areas (22.22%). Approximately one-third of both female and male groups report never consuming whole grains.

Thyroid dysfunction is the most common endocrine disorder associated with Down syndrome, with an estimated prevalence of 4–8% in newborns [58], as also confirmed by the participants in our survey. The consumption of whole grain products has a highly beneficial effect on plasma levels of free thyroxine (fT_4_), the biologically active form of the thyroid hormone T_4_. Unlike refined white flour products, which contain approximately five times the amount of simple carbohydrates, whole grains retain their outer bran layer, which is removed during the refining process. This outer layer is rich in dietary fiber and contains significant amounts of phytochemicals, antioxidants, and essential minerals such as selenium, iron, and zinc, all of which contribute to healthy thyroid function [59].

##### Milk and Dairy Products Consumption

As shown, there are no statistically significant differences in dietary habits between genders, with the exception of the consumption of fermented dairy products up to five times per week. In this case, girls show a statistically significantly higher preference for these products (37.50% girls, compared to 16.67% boys, *p* = 0.025).

Approximately half of the participants in both gender groups consume milk five times a week or more frequently, while 34.38% of female and 37.50% of male participants report never consuming milk. Further research would be necessary to explore the reasons for milk avoidance, especially considering that only one participant reported a cow’s milk allergy in the initial questionnaire. Unjustified elimination of foods should not be practiced in children with Down syndrome unless there is a clear medical indication. Studies have shown that some parents of children with Down syndrome restrict certain nutrients from their children’s diets without valid reasons. A notable example is the elimination of lactose. Research by Białek-Dratwa et al. [30] found that while only 19% of children were diagnosed with lactose intolerance, dietary restrictions were applied in 28.7% of cases without a confirmed diagnosis. A lactose-free diet is entirely unjustified in the absence of allergy, intolerance, or other metabolic or gastrointestinal disorders. Moreover, such unnecessary dietary restrictions can lead to the suppression of lactase enzyme production over time, which may, in turn, induce lactose intolerance [30].

A slightly lower percentage is observed in the case of fermented dairy product avoidance (15.6% of female and 23.6% of male participants), while nearly 44% of participants in both groups reported never consuming cheese. A significantly higher number of urban children consume milk once a day (*p* = 0.008), while a significantly higher number of rural children avoid milk (51.11% vs. 25.42%, *p* = 0.008). When examining the consumption of fermented dairy products, children from urban areas are more likely to consume them daily, i.e., five times per week or more, whereas children from rural areas more frequently consume these products only once a week (*p* = 0.005).

In general, cheese is consumed infrequently across all observed groups. Over 40% of participants reported not consuming cheese at all, while between 30% and 40% consume it once or twice per week.

##### Fruit and Vegetables Consumption

A total of 75% of female and 62.5% of male participants consume fruit on daily basis. On the other hand, in lower frequency categories like once or twice a week and never, male participants dominate (19.44% of male and 9.38% of female). There are observable differences in fruit consumption frequencies between males and females, meaning that fruit is generally more consumed by female participants, but none of these differences are statistically significant. In contrast to fruit, male participants consume vegetables more frequently on a daily basis (58.34% of males vs. 50% of females). Conversely, a higher proportion of female participants reported never consuming vegetables, 12.5% compared to 5.56% of males. As previously mentioned, families from rural areas produce more of their own fruit and vegetables compared to families from urban areas, who predominantly purchase them from grocery chains. However, this did not reflect in their consumption habits, as no statistically significant differences were found in the consumption of fruits and vegetables between these two groups.

Children with Down syndrome often show a preference for simple carbohydrates and foods that are easy to chew and swallow. This frequently leads to the rejection of fresh fruits and vegetables from their diet [27]. Possible reasons for this include a lack of parental knowledge about the benefits of proper nutrition, as well as the child’s aversion to the taste or texture of certain fruits and vegetables. A study by Białek-Dratwa et al. [30] confirmed that fruit and vegetable consumption is insufficient in the diets of children with Down syndrome. These findings are consistent with the results of our research. A comparative review of six studies of the eating habits of people with Down syndrome showed that people with Down syndrome tend to consume small amounts of vegetables and fruits, which leads to a reduced intake of fiber, antioxidants, and folic acid. This nutritional deficiency may contribute to a higher risk of non-communicable diseases such as diabetes, lipid metabolism disorders, and neurodegenerative conditions, which are already prevalent in this population due to genetic and metabolic factors. In addition, a diet low in vegetables can worsen common problems like constipation and being overweight, further compromising overall health [60].

##### Protein-Rich Food Consumption

Animal-derived proteins, such as those found in dairy products, eggs, poultry, and fish, are complete sources of protein that also provide essential vitamin B_12_, a deficiency of which can lead to macrocytic anemia [44]. Furthermore, the consumption of meat (including poultry and fish) enhances the absorption of both heme and non-heme iron in the same meal, as meat exerts a reducing effect on iron during digestion, thereby improving its bioavailability [61].

Male participants consume meat five times per week or more frequently than female participants (76.39% vs. 65.63%). This difference is most pronounced in the highest frequency category, two or more times per day, where no female participants reported such consumption, while 11.11% of male participants did. A *p*-value of 0.056 indicates a borderline statistical significance for this difference.

Research by Gruszka and Włodarek [60] indicated that individuals with Down syndrome most frequently select meat among protein-rich foods. Meat is an excellent source of complete proteins, iron, and vitamin B_12_, nutrients essential for the proper functioning of the human body. Given that vitamin B_12_ deficiency has been reported in individuals with Down syndrome, moderate meat consumption may be beneficial to their health. Among meat options, chicken, turkey, rabbit, lamb, and veal are considered the most nutritionally valuable. When consuming pork, it is advisable to select lean cuts such as the loin, due to the high saturated fat content found in fattier portions [44]. However, excessive intake of meat and consequently, saturated and omega-6 fatty acids may increase the risk of developing various health conditions, including cardiovascular disease and diabetes.

Low-frequency meat consumption (once a week) is more common among female participants, whereas approximately the same proportion of males and females, around 6%, reported never consuming meat. Similarly to fruits, although participants from rural areas more frequently produce their own meat rather than purchasing it, this did not result in a difference in the frequency of meat consumption between these rural and urban groups. Regarding processed meat products such as salami, sausages, and similar items, 78.13% of female and 75% of male participants reported consuming them once a week or less, suggesting relatively high awareness about the health implications of these products. No participants from rural areas consume processed meats two or more times per day, in contrast to 6.78% of participants from urban areas. However, a slightly higher percentage of urban residents never consume these products (52.54%), compared to participants from rural areas (46.67%), but observed differences do not reach statistical significance.

Unfortunately, 50% of female and 41.67% of male participants reported never consuming fish. When combined with those who consume it only once a week, the total reaches approximately 96% for both genders, which reflects a concerningly low level of fish intake. The same applies to the category of rural versus urban residence, with no differences observed between the groups. The reasons for such infrequent fish consumption may be varied and multifactorial. Fish is the richest natural source of omega-3 fatty acids and also provides high-quality protein, vitamins, and essential minerals. It is recommended that fish be consumed at least one to two times per week [62]. Despite their crucial functional role in brain tissue, the intake of omega-3 fatty acids is often inadequate among children and adults with Down syndrome. Essential fatty acids serve as precursors for biologically active compounds involved in inflammation and immune responses. They support neuronal development, synaptic membrane formation, signal processing, and neurotransmission. In addition, these fatty acids regulate gene expression in the brain [63,64]. Research using mouse models of Down syndrome has shown that fish oil, rich in omega-3 fatty acids, combined with other nutraceuticals, can moderately suppress the expression of the regulator calcineurin 1. These findings support the hypothesis that fish oil may serve as an effective and affordable intervention for genetically defined conditions, including cognitive impairments [62].

Egg consumption among participants in our study is also quite low, and the majority of both genders and both places of residence consume eggs once a week/rarely. Enriching the daily diet with eggs is beneficial to health, as eggs are a nutritionally balanced food, offering a favorable ratio of nutrients relative to their energy value. Research by Magenis et al. [44] involving 195 individuals with Down syndrome also revealed insufficient egg consumption among the majority of participants. This may be attributed to a lack of awareness regarding the health benefits of this nutrient-dense food. The same study revealed that while protein consumption was relatively frequent, it remained inadequate for more than half of the participants.

##### Oils and Fats, Nuts and Seeds Consumption

Approximately 90% of participants reported never consuming coconut oil, which is expected given the regional dietary patterns. However, it is concerning that around 46% of both male and female participants, over 51% of children from rural and 42% from urban areas never consume olive oil. Olive oil is consumed five times per week or more frequently by 37.50% of female and 40.28% of male participants. In general, the high frequencies of olive oil consumption among participants from urban areas are reported more often, although these differences are not statistically significant when compared to those from rural areas.

Unsaturated fatty acids, especially those found in cold-pressed oils, fish, and nuts, play a key role in reducing oxidative stress and contribute to the cognitive development of people with Down syndrome. Regular consumption of dietary sources rich in unsaturated fatty acids is associated with a lower risk of cardiovascular disease and obesity, both of which are common comorbidities in this population. Therefore, it is recommended to replace saturated and trans fats with vegetable oils and foods rich in unsaturated fats, as part of a comprehensive nutritional strategy aimed at preserving and improving the general health of people with Down syndrome [30].

Consumption of refined oils and saturated fats in high frequencies is reported by 40.63% of female and 30.56% of male participants. Additionally, participants from urban areas consume these products slightly more frequently, five times a week or more (38.98%), compared to participants from rural areas (26.67%). However, consumption once a week is more common among rural participants, although none of the observed frequencies reached statistical significance.

Over 80% of participants (gender and residence category) consume butter either once a week or never, which can be seen as a positive finding. Nevertheless, further education on the importance of unsaturated fatty acids, particularly for brain development and cognitive function, is needed. In this context, the consumption of nuts and seeds, foods of exceptionally high nutritional value, should also be considered. They are rich sources of unsaturated fats, dietary fiber, and essential minerals. Nuts are rich in tocopherol (vitamin E), phenolic compounds, and selenium nutrients with potent antioxidant properties. Given that oxidative stress is associated with both the development and the accelerated aging process observed in individuals with Down syndrome, regular nut consumption holds particular importance for this population. Several studies have shown that their regular intake may reduce the risk of certain diseases and also contribute to improving cognitive development [65,66].

Unfortunately, over 50% of participants in our study, of both genders, reported never consuming nuts, while more than 30% consume nuts only once a week, and just over 20% consume seeds with the same frequency. In addition, 68% of children from rural areas and 54% from urban areas avoid seeds. Research by Białek-Dratwa et al. [30] also revealed insufficient consumption of nuts by the majority (72.31%) of children and adults with Down syndrome, without any medically justified reason.

##### Fast Food, Sweets, and Snack Consumption

Children with Down syndrome often have increased carbohydrate and caloric intake, with a tendency to consume high-energy foods such as pasta, bread, sweets, and sugary beverages. Conversely, their intake of essential nutrients like calcium, certain B vitamins, and water is often insufficient [44].

In addition, among those with sensory processing difficulties, particularly texture sensitivity, there may be a preference for foods that are soluble, crunchy, or salty. These sensory-driven preferences can lead to a more limited diet, increased snacking, and a higher risk of nutritional deficiencies [18].

It is encouraging that over 80% of participants in our study of all genders and places of residence reported never consuming fast food, and only 15% consume this type of food once a week. Notably, female participants never reported consuming fast food more frequently than once a week, whereas among male participants, one individual from a rural area reported consuming it two or more times per day and once per day.

Approximately 15–20% of participants of both genders reported daily consumption of sweets and snack foods, whereas 30–40% indicated that they never consume such items. An exception was observed among female participants, 25% of whom reported complete avoidance of sweets. Children from rural areas eat sweets and snacks more frequently than children from urban areas, but no statistically significant difference was confirmed.

##### Liquids Consumption

When comparing the consumption of various beverages, water was most frequently reported as being consumed two or more times per day. In contrast, only 5–12% of respondents of both genders reported consuming tea, natural juices, or carbonated beverages with the same frequency (two or more times a day), with carbonated drinks being the least frequently consumed in this category (5–6%). Carbonated drinks are more often avoided by rural children (84.44%) than by urban children (77.97%), but both numbers are high and do not exhibit a statistical difference. A concerning finding is that 50% of female and 41.67% of male respondents reported never consuming natural juices, with similar percentages for both rural and urban places of residence. However, more children from rural areas (17.78%) than from urban areas (6.78%) consume natural juice twice a day. These beverages could represent a valuable source of vitamins and minerals, particularly for children who experience difficulties chewing solid food or who refuse to eat fruit, as noted by parents in the initial survey. Furthermore, 53% of female and 69.44% of male respondents stated that they never consume tea. Consumption of carbonated beverages five times a week or more often was reported by only two female and 10 male participants. Interestingly, 20% of participants from rural areas never consume water, compared to 1.69% of participants from urban areas (*p* = 0.002). A deficit in water consumption has been observed in individuals with Down syndrome, with parental influence playing a significant role in shaping their children’s hydration habits. Since many children dislike the “tastelessness” of water and often refuse to drink it, parents may resort to offering juices or other flavored beverages that are more appealing to the child, often without considering the potential long-term health consequences of such choices. Alternative sources of hydration, such as unsweetened tea or vegetable juice, can be beneficial as they not only contribute to fluid intake but also provide additional vitamins and minerals [30]. Children are particularly vulnerable to dehydration due to their higher body surface area-to-volume ratio, which makes them more sensitive to environmental temperature changes. Moreover, especially at a young age, children’s access to fluids is largely dependent on their parents or caregivers, which may limit their overall intake. Inadequate water consumption in children can also negatively impact cognitive performance. Studies conducted across European countries have shown that over 80% of children consume less water than the recommended daily intake [67,68].

#### 3.3.2. Analysis of Respondents’ Dietary Habits in Relation to Parental Educational Attainment

Table 4 illustrates the relationship between parental educational attainment and the dietary habits of the respondents. A total of 55 mothers and 72 fathers have completed secondary education, while 49 mothers and 32 fathers hold a university degree. It is evident that the mother’s level of education has a substantially greater impact on children’s dietary habits compared to the father’s educational level. This finding is consistent with the fact that, in the Republic of Croatia, the mother is still predominantly responsible for meal planning and preparation within the household, regardless of education and employment.

There is strong evidence of an association between the educational attainment of parents, particularly mothers, and children’s eating behavior. The INPACT study conducted in the Netherlands found that children of mothers with higher levels of education consumed more fruit and vegetables and were more likely to eat breakfast regularly. These associations were mediated by factors within the home food environment, including parental dietary practices and the availability of healthy food options [69]. Similarly, results from the IDEFICS study, which included children from eight European countries, found that lower parental education was associated with increased consumption of foods high in sugar and fat, such as French fries and sugar-sweetened beverages. In contrast, higher parental educational attainment was positively correlated with higher intakes of fruit, vegetables, and wholemeal bread [70].

##### Carbohydrate-Rich Foods Consumption

Within the food rich in carbohydrates, it is evident that children of mothers with secondary education consume white bread on a daily basis at a statistically significantly (*p* = 0.032) higher rate (30.91%) compared to children of mothers with a university degree (12.24%). This finding suggests that maternal educational attainment plays a key role in shaping children’s dietary habits, with those having lower levels of education more likely to provide highly refined carbohydrate-rich foods such as white bread. Additionally, it can be observed that a higher proportion of children whose mothers have completed secondary education never or rarely consume whole grain bread and cereals. This further reinforces the idea that the education level of the mother may influence not only the choice of foods but also the variety and nutritional quality of the diet provided to children. Conversely, a higher proportion of children whose mothers and fathers hold a university degree reported never consuming white bread, although these differences were not found to be statistically significant. This suggests that while higher parental education may lead to healthier dietary choices, these habits may not be as pronounced in the consumption of specific foods such as white bread. It is important to note that while the statistical significance is not present in some cases, the trends observed indicate potential areas for further investigation, particularly in terms of parental influence and socio-economic factors related to education.

Although children of mothers with secondary education consume potatoes five times a week or more frequently, more often than children of mothers with university education, this difference is not statistically significant. However, children of mothers with university education consume potatoes significantly more often once a week compared to children of mothers with secondary education (87.76% vs. 67.27%, *p* = 0.019). Children of mothers with higher education tend to consume fewer carbohydrate-rich foods, such as white bread and potatoes. This may be attributed to several factors. Firstly, mothers with higher education are generally more aware of the impact of nutrition on health, leading them to choose healthier, more balanced diets for their children. Additionally, higher educational attainment is often linked to higher socio-economic status, which may provide better access to healthier food options.

##### Milk and Dairy Products Consumption

Milk is consumed on a daily basis significantly more by children whose parents have completed secondary education, with a statistically significant difference observed among fathers for the frequency of consumption two or more times per day (*p* = 0.002). In contrast, for mothers, this frequency showed borderline significance (*p* = 0.062). On the other hand, children whose parents hold university degrees consume milk significantly more often, five times a week, with this finding also statistically confirmed for mothers (*p* = 0.048). The level of paternal education generally demonstrates a statistically significant impact on milk consumption. A high frequency of consuming fermented dairy products on a daily basis is more commonly observed among children of mothers with secondary education, although this difference is not statistically significant. However, children of mothers with higher education consume these products significantly more frequently on a weekly basis (*p* = 0.018). As previously stated, many participants do not consume cheese, and there is not any statistical difference between groups in the case of parents’ education level.

##### Fruit and Vegetable Consumption

Children of parents with higher educational attainment are more likely to consume fruit two or more times per day, as well as vegetables. However, these differences do not reach statistical significance. The same can be observed for vegetable consumption once per day and five times per week. A borderline statistical significance is in the case of vegetable consumption once per week (16.36% of children of mothers with secondary education versus 4.08% of children of mothers with higher education), with a *p*-value of 0.056, and in the case of non-consumption of vegetables, with a *p*-value of 0.063 (12.73% of children of mothers with secondary education versus 2.04% of children of mothers with higher education). Globally, mothers’ education is statistically confirmed as significant on vegetable consumption by Fisher’s test with Monte Carlo simulation (*p* = 0.031) and by chi-square test (*p* = 0.033), while fathers’ education level does not exhibit statistical significance.

##### Protein-Rich Food Consumption

Parental education, both maternal and paternal, significantly influences the consumption of meat and processed meat products. Children of highly educated parents are more likely to consume meat at least five times per week. A statistically significant difference was observed for the frequency of consumption up to five times per week (*p* = 0.028) among mothers. When examining the frequency of consumption once per week, the results were reversed, with children of mothers (*p* = 0.026) and fathers with secondary education displaying higher frequencies of consumption, as well as for the rare or never consumption category. In contrast, the pattern for processed meat products differs from that of meat. Daily consumption of processed meat products is more common among children of parents with secondary education, while avoidance of such products is more frequent among children of highly educated parents. This finding is consistent with the previously established pattern that the level of education correlates with socio-economic status, influencing purchasing decisions, particularly in terms of more expensive meats and less expensive processed meat products. A statistically significant global difference in the consumption of processed meat products was confirmed according to educational level, both for mothers (*p* = 0.008) and fathers (*p* = 0.046). Interestingly, for fathers, no individual consumption frequency showed statistical significance; however, a global effect was confirmed as a result of cumulative effects, where small associations across multiple rows collectively yielded a significant overall result.

Although no statistically significant differences were observed in fish consumption between groups based on parental education level, and overall results indicate equally low consumption in both categories and observed groups when it comes to daily intake, it is nevertheless evident that fish is more frequently consumed five times per week and once per week by children of university educated mothers and fathers. Conversely, never consuming fish is more common among children of parents with secondary education. Regarding egg consumption, intake five times per week is notably higher among children of highly educated mothers. While the difference does not reach conventional levels of statistical significance, it approaches the threshold (14.29% vs. 3.64%, *p* = 0.08), indicating a potential trend worth further investigation.

##### Oils and Fats, Nuts and Seeds Consumption

The influence of maternal education on the consumption of olive oil is statistically significant at the global level (*p* = 0.003), confirmed by Fisher’s test and the chi-square test. Mother’s education is also significant for specific consumption frequencies—up to five times per week (*p* = 0.005) and never (*p* = 0.0008). Paternal education also demonstrates global significance (*p* = 0.039). Children whose parents have a university level education tend to consume olive oil more frequently, while those whose parents have completed only secondary education are more likely to never consume it. A similar pattern can be observed in the consumption of nuts and seeds, which is statistically confirmed in the case of exclusion of nuts from the diet (*p* = 0.029). This practice is more prevalent among mothers with secondary education (62.27%) compared to those with higher education (44.90%). Consistent with previously stated regarding the consumption of higher-cost products, a higher level of education among both mothers and fathers is associated with more frequent inclusion of olive oil in children’s diets. Conversely, lower educational attainment corresponds with more frequent exclusion of olive oil from the diet. A similar trend is observed for coconut oil, although its use is generally rare across both groups. Nevertheless, paternal education was found to have a statistically significant global impact on the frequency of coconut oil consumption (*p* = 0.014).

No clear pattern or statistical significance was observed in the comparison of refined oil and saturated fat consumption. However, in the case of butter, a trend can be noted whereby children of highly educated mothers and fathers consume butter more frequently, while its exclusion from the diet is more common among children of parents with secondary education. Nevertheless, these differences do not reach statistical significance.

##### Fast Food, Sweets, and Snack Consumption

Higher consumption of fast food, sweets, and salty snacks is observed among children whose mothers and fathers have secondary education. However, a statistically significant difference was confirmed only in specific cases: for the never consumption frequency of sweets among mothers, where 44.90% of children of highly educated mothers never consume sweets compared to 25.45% of children of mothers with secondary education; as well as for the global consumption of salty snacks (*p* = 0.040), confirmed by chi-square and Fisher’s test and for the frequency of consumption two or more times per day (*p* = 0.028) of salty snacks.

##### Liquids Consumption

Children of highly educated parents consume carbonated beverages more frequently across all consumption categories, although no statistically significant differences were found between the groups. Natural juice consumption once per week is more common among children of highly educated mothers and fathers, while daily consumption, two or more times per day, is more frequent among children of highly educated fathers and mothers with secondary education, similar to the children from rural areas. However, these differences are not statistically significant, nor are those observed for tea and water consumption. Nevertheless, 12.73% of mothers with lower educational attainment reported that their children never consume water, compared to 6.12% of mothers with higher education, with a similar trend observed among fathers.

#### 3.3.3. Analysis of Respondents’ Dietary Habits in Relation to Mother’s Employment Status and Monthly Financial Expenditure on Food

Table 5 represents the relationship between mother’s employment status as well as share of food expenditures in family income on the dietary habits of the respondents

A total of 39 mothers were unemployed, while 65 were employed. The influence of fathers’ employment status was not analyzed in this context, as only 5 out of the 104 fathers were unemployed.

##### Carbohydrate-Rich Foods Consumption

Daily consumption of white bread was more prevalent among children of unemployed mothers (58.97%) compared to those whose mothers were employed (30.77%). A similar pattern was observed among children from households in which monthly food expenditure exceeded 50% of total income. A statistically significant difference was observed in the frequency of consuming white bread two or more times per day in relation to maternal employment status (*p* = 0.009) and monthly food expenditures (*p* = 0.035), with unemployment and higher food expenditures being associated with increased consumption of white bread. The global influence of mothers’ employment status was confirmed statistically by Fisher’s test (*p* = 0.01) and the chi-square test. When examining the frequencies of less frequent consumption (once a week or never), higher percentages were found among children of employed mothers and children from households that spend less than 50% of their income on food.

The trend is reversed in the case of consuming dark/whole grain bread, where maternal employment and a lower proportion of food expenditures in monthly family income are associated with higher consumption of this type of bread, although the difference is not statistically significant. Additionally, the consumption of whole grains five times a week is statistically significantly more frequent in families where food expenditures do not exceed 50% of monthly income (*p* = 0.049), while daily consumption of whole grains shows the opposite trend. A higher frequency of consumption of potatoes, pasta, and rice (five times a week or more) is observed among children of unemployed mothers and those from families with higher food expenditures within the household budget, whereas a lower frequency of consumption shows the opposite trend. This finding was statistically confirmed globally for potato consumption (*p* = 0.019), and for the impact of family food expenditures on the frequency of rice consumption once or twice a week. Among families with food expenditures less than 50%, 79.71% of children consume rice once a week, whereas among families with higher expenditures, only 57.14% of children consume rice once a week. The observed trend supports the notion that a mother’s employment status and the family’s income may impact not only the selection of foods but also the diversity and nutritional value of the diet offered to children.

Evidence from studies conducted in Australia, the United States, and Japan highlights the complex relationship between maternal employment, household income, and children’s dietary behaviors. In Australia, lower household income and maternal employment of 21–35 h per week were linked to greater consumption of energy-dense, nutrient-poor foods among children [36]. A U.S. study similarly found that increased maternal work hours were associated with higher intake of unhealthy foods, lower intake of healthy foods, greater screen time, and elevated BMI, particularly among children from higher socioeconomic backgrounds [34]. In Japan, longer maternal working hours were associated with higher intake of white rice and increased body mass index in children, though no significant associations were found with vegetable or sweetened beverage intake. The study emphasized the influence of factors such as nutrition knowledge, dietary attitudes, and family environment on children’s eating habits [71]. Collectively, these findings suggest that both structural and psychosocial factors must be considered when addressing childhood nutrition and health.

##### Milk and Dairy Products Consumption

Unemployment of mothers and higher food expenditures within the family budget support the higher frequencies of milk consumption, although no statistically significant differences between groups were observed. However, children of unemployed mothers consume fermented dairy products twice a day significantly more often (*p* = 0.006) than children of unemployed mothers (20.51% vs. 3.08%). A correlation between mothers’ employment status and the consumption of fermented dairy products has also been statistically confirmed by Fisher’s test (*p* = 0.04) and the chi-square test.

Lower socio-economic status (unemployment and high financial expenditures on food within family income) is associated with high frequencies of milk consumption, as well as fermented dairy products. This trend has also been noted in relation to the parents’ level of education, as previously discussed in Table 4.

As previously mentioned, cheese is generally consumed by the majority of respondents only once a week or less frequently or is entirely avoided. However, a pattern can be observed where children of unemployed mothers consume cheese more frequently, about once a day, while children of employed mothers consume it five times a week. Nevertheless, no statistically significant differences were found in any consumption frequency.

##### Fruits and Vegetables Consumption

Children of unemployed mothers and those from lower-income families consume fruit two or more times per day more frequently than children of employed mothers and those from higher-income households. However, this difference is not statistically significant. On the other hand, daily fruit consumption, as well as consumption five times per week, is more common among children of employed mothers and in families that allocate less than 50% of their monthly income to food, with the difference being statistically significant (*p* = 0.032). Additionally, low frequencies of fruit and vegetable consumption are more frequently observed among children of unemployed mothers and in families whose monthly food expenditures exceed 50% of their income.

##### Protein-Rich Food Consumption

The consumption of meat and processed meat products at least twice daily is more prevalent among children of unemployed mothers and families that allocate a larger proportion of their monthly income to food. However, when considering the frequency of meat consumption once per day or five times per week, a higher intake is observed among children of employed mothers and those from families spending less than 50% of their monthly income on food (*p* = 0.0002). Fish consumption is generally low across both categories (mother’s employment and share of food costs in family income) and all groups; however, it is higher among children of employed mothers and families that allocate less than 50% of their monthly income to food. The highest frequency of meat and processed meat consumption, twice per day, is observed among children of unemployed mothers; this same group records the lowest fish intake. These two patterns appear to be interrelated, suggesting that while families may consume substantial amounts of protein-rich foods, fish, an important source not only of high-quality proteins but also of essential micronutrients such as omega-3 fatty acids, is more closely associated with higher socioeconomic status.

Maternal employment status has a statistically significant effect on egg consumption (*p* = 0.02), with higher consumption frequencies, once per week or more, being more common among children of unemployed mothers. Conversely, lower consumption frequency of eggs, specifically once per week, is significantly more prevalent among children of employed mothers (*p* = 0.015).

##### Oil, Fat, Nut, and Seed Consumption

When examining individual consumption frequencies of olive oil, a statistically significant difference was observed for the frequency two or more times per day (*p* = 0.043) corresponding to 10.77% of children of employed mothers, who reported consuming olive oil at this specific frequency, while none of the children of unemployed mothers reported the same. Although other individual frequency categories did not reach statistical significance, children of employed mothers tended to consume olive oil more frequently, at five times per week. In contrast, once per day, once per week, and never consumption frequencies were more common among children of unemployed mothers. Overall, lower consumption frequencies were more prevalent in the group of unemployed mothers.

Very few participants reported consuming coconut oil; however, the majority of those who do consume it belong to the group of children of employed mothers and families that allocate less than 50% of their monthly budget to food. Although no statistically significant differences were observed between the groups, a clear trend of higher consumption of refined oils and saturated fats was noted among children of unemployed mothers and families whose monthly food expenditures exceed 50% of their total income. Maternal employment status also had a statistically significant effect on butter consumption (*p* = 0.02). However, when examining specific frequency categories of butter intake, children of employed mothers consumed butter significantly more often than children of unemployed mothers in the frequency category up to five times per week (15.38% vs. 0%). Conversely, in the once per week frequency category, butter consumption was more prevalent among children of unemployed mothers (38.46% vs. 21.54%).

Family financial expenditure on food (*p* = 0.001) as well as maternal employment status (*p* = 0.01) have a significant impact on the consumption of nuts. This effect is further supported by significant differences in specific frequencies, including once per day (*p* = 0.012) and never frequencies (*p* = 0.008) within the maternal employment status, and once per week (*p* = 0.004) and never (*p* = 0.0008) within the category of monthly food expenditures. The trend observed is that high frequencies of nuts consumption are more prevalent among children of employed mothers and families whose monthly food expenditures do not exceed 50% of their income, whereas avoidance of consumption is more common among children of unemployed mothers and families whose monthly food expenditures exceed 50% of their income. A similar trend is evident in seed consumption, although no statistically significant differences were found between the groups under observation.

##### Fast Food, Sweets, and Snack Consumption

Only two respondents consume fast food on a daily basis, at least once per day, and both belong to the group of children of unemployed mothers. This is contrary to the expectation that such a diet would be more frequently adopted by employed mothers, who have less time to prepare meals. However, the small sample size within this frequency limits the ability to draw definitive conclusions. A study examining German families found that maternal employment was linked to reduced time spent by mothers on meal preparation and less time spent eating with their children at home. Conversely, maternal employment was associated with increased time spent on joint meals away from home. These changes in time allocation may influence children’s dietary habits [72].

When examining consumption habits for other food items in this food category, the employment status of mothers was found to have a statistically significant effect on the consumption of salty snacks (*p* = 0.016), specifically in the frequency category of two or more times a day (*p* = 0.002). In this category, 15.38% of children of unemployed mothers consumed snacks, whereas no children of employed mothers reported consuming snacks at this frequency. Low-frequency consumption of snacks was more common among children of employed mothers. Similarly, among children from families where monthly food expenditures exceed 50% of monthly income, a statistically significantly more often consumption was observed for those consuming snacks once per day (*p* = 0.035). Additionally, sweets were consumed significantly more often by children of unemployed mothers in the frequency of two or more times per day (*p* = 0.05). Thus, maternal unemployment does not appear to contribute to a greater focus on healthier dietary options, but rather to the opposite effect.

##### Liquids Consumption

Monthly family financial expenditures on food influence the frequency of water consumption borderline significantly (*p* = 0.058), with a significant difference observed in the individual frequency category of “once per week” (*p* = 0.036). Specifically, 8.57% of children from families whose monthly food expenditures exceed 50% of their income consume water only once per week, while no children from families whose food expenditures are below 50% of their income report consuming water at this frequency. The majority of children across all categories consume water two or more times per day (over 70%), although high frequencies of consumption are more prevalent among children of employed mothers and children from families where monthly food expenditures do not exceed 50% of income.

Tea consumption at high frequencies predominates among children of unemployed mothers, but further inquiry is needed regarding the type of tea and methods of sweetening to draw a definitive conclusion about the benefits of tea consumption for children in this study. No statistically significant differences were observed between the groups, as was the case with natural juices and carbonated beverages. However, the consumption of natural juices is higher among children from families whose food expenditures account for less than 50% of their income and among children of employed mothers, likely due to the cost of such beverages. Carbonated drinks are more frequently avoided by children of employed mothers and families with higher incomes, whereas high-frequency consumption of carbonated drinks is somewhat more common among children of unemployed mothers and families with lower incomes, although no statistically significant difference was found between the groups.

In summary, this study highlights the multifaceted nutritional challenges faced by children with Down syndrome, including a notable prevalence of overweight and obesity alongside feeding difficulties and comorbidities such as congenital heart defects, hypothyroidism, and gastrointestinal anomalies. Feeding problems, such as delayed chewing, refusal of certain food textures, and oral-motor dysfunction, were common and often influenced by both medical and emotional factors. Many parents face considerable stress and uncertainty, which can lead to cautious or restrictive feeding practices.

These findings underscore the urgent need for tailored nutritional guidance, early intervention, and coordinated support involving healthcare providers, educators, and policymakers. Empowering parents and caregivers through targeted education and practical strategies, focusing on safe feeding techniques, oral-motor skill development, and positive mealtime experiences, is essential to improve dietary habits and overall health outcomes in this vulnerable population. Raising public awareness and integrating specialized support into existing health and educational systems will help bridge current gaps and foster a more nutritionally supportive environment for children with Down syndrome.

## 4. Conclusions

This study highlights the significant impact of socioeconomic factors and place of residence on the dietary habits and nutritional status of children with Down syndrome. Children from lower socioeconomic backgrounds—particularly those with unemployed or less-educated mothers and families spending over 50% of their income on food—demonstrated poorer dietary patterns, including higher consumption of refined carbohydrates and lower intake of nutrient-dense foods such as whole grains, fish, and vegetables.

Although rural families often produce their own food, this did not translate into healthier dietary habits, indicating that food access alone is insufficient without appropriate nutritional knowledge. Urban residence and higher maternal education were associated with more favorable dietary behaviors.

The findings underscore the need for targeted nutritional interventions and education, particularly in rural and low-income households. Addressing these socioeconomic disparities is essential for improving dietary quality and long-term health outcomes in children with Down syndrome.

## Figures and Tables

**Table 1:** Respondents weight-for-age percentile according to growth charts for children with Down syndrome [38].

Age	n (F)	Average Percentile Weight-for-Age F	n (F) < 5th Percentile	n (F) 85th –94th Percentile	n (F) > 95th Percentile	n (M)	Average Percentile Weight-for-Age M	n (M) < 5th Percentile	n (M) 85th –94th Percentile	n (M) > 95th Percentile
6–11 months	1	80	0	0	0	6	50	0	1 (16.7%)	0
1–3 years	17	56	1 (5.9%)	3 (17.6%)	0	24	61	3 (12.5%)	4 (16.7%)	4 (16.7%)
4–7 years	5	62	0	1 (20%)	0	23	69	2 (8.7%)	5 (21.7%)	3 (13.1%)
8–15 years	6	75	0	2 (33.3%)	0	17	62	0	3 (17.6%)	2 (11.8%)
16–19 years	3	48	0	0	0	2	52	0	0	0

n—number of participants; F—female; M—male.

**Table 2:** The relationship between urban and rural living environments and grocery shopping habits.

		Single Answers Only				All Answers			
Question	Answer	Urban (n = 59)	Rural (n = 45)	*p*	*p* Global	Urban (n = 59)	Rural (n = 45)	*p*	*p* Global
Where do you most often buy fresh fruits and vegetables?	At the local market	14	6	0.176	0.293	33	14	0.07	0.084
	In large retail chains	12	10	1.000		22	17	0.849	
	Directly from the manufacturer	2	1	1.000		12	6	0.61	
	I don’t buy. We produce ourselves	8	12	0.113		17	22	0.035	
Where do you most often buy fresh meat?	At the local market	8	8	0.589	0.052	19	11	0.529	0.015
	In large retail chains	21	7	0.019		28	11	0.034	
	Directly from the manufacturer	13	12	0.637		21	19	0.448	
	I don’t buy. We produce ourselves	4	9	0.067		5	13	0.009	
Where do you most often buy other food?	In small specialty stores	8	14	0.051	0.051				
	In large retail chains	51	31	0.051					

Statistical significance *p* < 0.05.

**Table 3:** Dietary habits of respondents in relation to gender and place of residence.

Question		Gender						Place of Residence					
How Often Does Your Child with Down Syndrome Consume:	Frequency of Consumption	F (n = 32)	M (n = 72)	F (%)	M (%)	*p*	*p* Global	U (n = 59)	R (n = 45)	U (%)	R (%)	*p*	*p* Global
White/semi-white bread	Two or more times a day	6	14	18.75	19.44	1.000	0.797	7	13	11.86	28.89	0.043	0.162
	Once a day	8	15	25.00	20.83	0.620		15	8	25.42	17.78	0.475	
	Up to five times a week	3	6	9.38	8.33	1.000		6	3	10.17	6.67	0.729	
	Once or twice a week	6	9	18.75	12.50	0.550		11	4	18.64	8.89	0.260	
	Rarely/never	9	28	28.13	38.89	0.380		20	17	33.90	37.78	0.686	
Black/wholemeal bread	Two or more times a day	3	3	9.38	4.17	0.370	0.810	5	1	8.47	2.22	0.231	0.295
	Once a day	2	8	6.25	11.11	0.720		5	5	8.47	11.11	0.743	
	Up to five times a week	2	5	6.25	6.94	1.000		4	3	6.78	6.67	1.000	
	Once or twice a week	5	11	15.63	15.28	1.000		12	4	20.34	8.89	0.169	
	Rarely/never	20	45	62.50	62.50	1.000		33	32	55.93	71.11	0.153	
Whole grains such as barley, oats, etc., in the form of grains, groats, or flour	Two or more times a day	2	8	6.25	11.11	0.720	0.807	4	6	6.78	13.33	0.323	0.225
	Once a day	11	17	34.38	23.61	0.340		20	8	33.90	17.78	0.078	
	Up to five times a week	2	6	6.25	8.33	1.000		6	2	10.17	4.44	0.461	
	Once or twice a week	6	17	18.75	23.61	0.800		12	11	20.34	24.44	0.641	
	Rarely/never	11	24	34.38	33.33	1.000		17	18	28.81	40.00	0.296	
Potatoes	Two or more times a day	1	2	3.13	2.78	1.000	0.291	0	3	0.00	6.67	0.078	0.069
	once a day	0	3	0.00	4.17	0.550		0	3	0.00	6.67	0.078	
	Up to five times a week	0	7	0.00	9.72	0.100		4	3	6.78	6.67	1.000	
	Once or twice a week	27	53	84.38	73.61	0.310		49	31	83.05	68.89	0.104	
	Rarely/never	4	7	12.50	9.72	0.730		6	5	10.17	11.11	1.000	
Pasta	Two or more times a day	1	1	3.13	1.39	0.520	0.839	0	2	0.00	4.44	0.185	0.105
	Once a day	0	2	0.00	2.78	1.000		0	2	0.00	4.44	0.185	
	Up to five times a week	1	2	3.13	2.78	1.000		3	0	5.08	0.00	0.256	
	Once or twice a week	22	53	68.75	73.61	0.640		44	31	74.58	68.89	0.659	
	Rarely/never	8	14	25.00	19.44	0.600		12	10	20.34	22.22	0.814	
Rice	Two or more times a day	0	3	0.00	4.17	0.550	0.841	1	2	1.69	4.44	0.577	0.650
	Once a day	2	1	6.25	1.39	0.220		1	2	1.69	4.44	0.577	
	Up to five times a week	0	0	0.00	0.00	n/a		0	0	0.00	0.00	n/a	
	Once or twice a week	23	52	71.88	72.22	1.000		45	30	76.27	66.67	0.378	
	Rarely/never	7	16	21.88	22.22	1.000		12	11	20.34	24.44	0.641	
Milk	Two or more times a day	5	11	15.63	15.28	1.000	0.839	9	7	15.25	15.56	1.000	0.022
	Once a day	6	16	18.75	22.22	0.800		18	4	30.51	8.89	0.008	
	Up to five times a week	5	10	15.63	13.89	0.770		8	7	13.56	15.56	0.786	
	Once or twice a week	5	8	15.63	11.11	0.530		9	4	15.25	8.89	0.384	
	Rarely/never	11	27	34.38	37.50	0.830		15	23	25.42	51.11	0.008	
Fermented dairy products (yogurt, cream, kefir, etc.)	Two or more times a day	2	8	6.25	11.11	0.720	0.076	5	5	8.47	11.11	0.743	0.053
	Once a day	4	21	12.50	29.17	0.084		13	12	22.03	26.67	0.647	
	Up to five times a week	12	12	37.50	16.67	0.025		10	14	16.95	31.11	0.104	
	Once or twice a week	9	14	28.13	19.44	0.440		19	4	32.20	8.89	0.005	
	Rarely/never	5	17	15.63	23.61	0.440		12	10	20.34	22.22	0.814	
Cheese	Two or more times a day	0	0	0.00	0.00	n/a	0.516	0	0	0.00	0.00	n/a	0.306
	Once a day	2	6	6.25	8.33	1.000		4	4	6.78	8.89	0.724	
	Up to five times a week	1	8	3.13	11.11	0.270		3	6	5.08	13.33	0.171	
	Once or twice a week	15	26	46.88	36.11	0.390		27	14	45.76	31.11	0.158	
	Rarely/never	14	32	43.75	44.44	1.000		25	21	42.37	46.67	0.694	
Fruit	Two or more times a day	8	21	25.00	29.17	0.810	0.607	14	15	23.73	33.33	0.378	0.484
	Once a day	16	24	50.00	33.33	0.130		26	14	44.07	31.11	0.314	
	Up to five times a week	5	13	15.63	18.06	1.000		10	8	16.95	17.78	1.000	
	Once or twice a week	2	9	6.25	12.50	0.500		7	4	11.86	8.89	0.753	
	Rarely/never	1	5	3.13	6.94	0.660		2	4	3.39	8.89	0.399	
Vegetables	Two or more times a day	4	13	12.50	18.06	0.575	0.748	7	10	11.86	22.22	0.187	0.309
	Once a day	12	29	37.50	40.28	0.831		26	15	44.07	33.33	0.314	
	Up to five times a week	9	18	28.13	25.00	0.810		17	10	28.81	22.22	0.504	
	Once or twice a week	3	8	9.38	11.11	1.000		4	7	6.78	15.56	0.201	
	Rarely/never	4	4	12.50	5.56	0.247		5	3	8.47	6.67	1.000	
Meat	Two or more times a day	0	8	0.00	11.11	0.056	0.263	4	4	6.78	8.89	0.724	0.583
	Once a day	9	18	28.13	25.00	0.810		15	12	25.42	26.67	1.000	
	Up to five times a week	12	29	37.50	40.28	0.831		27	14	45.76	31.11	0.158	
	Once or twice a week	9	12	28.13	16.67	0.195		10	11	16.95	24.44	0.460	
	Rarely/never	2	5	6.25	6.94	1.000		3	4	5.08	8.89	0.463	
Meat products (salami, hot dogs, pâté, etc.)	Two or more times a day	1	3	3.13	4.17	1.000	0.989	4	0	6.78	0.00	0.131	0.225
	Once a day	3	6	9.38	8.33	1.000		5	4	8.47	8.89	1.000	
	Up to five times a week	3	9	9.38	12.50	0.751		4	8	6.78	17.78	0.121	
	Once or twice a week	9	18	28.13	25.00	0.810		15	12	25.42	26.67	1.000	
	Rarely/never	16	36	50.00	50.00	1.000		31	21	52.54	46.67	0.694	
Eggs	Two or more times a day	0	2	0.00	2.78	1.000	0.790	0	2	0.00	4.44	0.185	0.548
	Once a day	1	4	3.13	5.56	1.000		2	3	3.39	6.67	0.650	
	Up to five times a week	2	7	6.25	9.72	0.718		5	4	8.47	8.89	1.000	
	Once or twice a week	19	33	59.38	45.83	0.288		30	22	50.85	48.89	1.000	
	Rarely/never	10	26	31.25	36.11	0.663		22	14	37.29	31.11	0.540	
Fish	Two or more times a day	0	0	0.00	0.00	n/a	0.788	0	0	0.00	0.00	n/a	0.929
	once a day	0	0	0.00	0.00	n/a		0	0	0.00	0.00	n/a	
	Up to five times a week	1	2	3.13	2.78	1.000		2	1	3.39	2.22	1.000	
	Once or twice a week	15	40	46.88	55.56	0.524		32	23	54.24	51.11	0.844	
	Rarely/never	16	30	50.00	41.67	0.522		25	21	42.37	46.67	0.694	
Olive oil	Two or more times a day	1	6	3.13	8.33	0.430	0.829	3	4	5.08	8.89	0.463	0.730
	Once a day	5	13	15.63	18.06	1.000		11	7	18.64	15.56	0.796	
	Up to five times a week	6	10	18.75	13.89	0.560		11	5	18.64	11.11	0.412	
	Once or twice a week	5	10	15.63	13.89	0.770		9	6	15.25	13.33	1.000	
	Rarely/never	15	33	46.88	45.83	1.000		25	23	42.37	51.11	0.430	
Coconut oil	Two or more times a day	0	0	0.00	0.00	n/a	0.456	0	0	0.00	0.00	n/a	0.469
	Once a day	0	1	0.00	1.39	1.000		0	1	0.00	2.22	0.433	
	Up to five times a week	0	2	0.00	2.78	1.000		2	0	3.39	0.00	0.504	
	Once or twice a week	4	4	12.50	5.56	2.320		4	4	6.78	8.89	0.724	
	rarely/never	28	65	87.50	90.28	0.430		53	40	89.83	88.89	1.000	
Refined oils and saturated fats (sunflower oil, canola oil, lard, etc.)	Two or more times a day	0	2	0.00	2.78	1.000	0.554	1	1	1.69	2.22	1.000	0.239
	Once a day	6	9	18.75	12.50	0.546		12	3	20.34	6.67	0.055	
	Up to five times a week	7	11	21.88	15.28	0.413		10	8	16.95	17.78	1.000	
	Once or twice a week	8	19	25.00	26.39	1.000		12	15	20.34	33.33	0.176	
	Rarely/never	11	31	34.38	43.06	0.517		24	18	40.68	40.00	1.000	
Butter	Two or more times a day	0	0	0.00	0.00	n/a	0.267	0	0	0.00	0.00	n/a	0.487
	Once a day	2	4	6.25	5.56	1.000		3	3	5.08	6.67	1.000	
	Up to five times a week	2	8	6.25	11.11	0.720		8	2	13.56	4.44	0.181	
	Once or twice a week	13	16	40.63	22.22	0.062		16	13	27.12	28.89	1.000	
	Rarely/never	15	44	46.88	61.11	0.202		32	27	54.24	60.00	0.690	
Fast food (burgers, pizza, etc.)	Two or more times a day	0	1	0.00	1.39	1.000	1.000	0	1	0.00	2.22	0.433	0.641
	Once a day	0	1	0.00	1.39	1.000		0	1	0.00	2.22	0.433	
	Up to five times a week	0	1	0.00	1.39	1.000		1	0	1.69	0.00	1.000	
	Once or twice a week	5	11	15.63	15.28	1.000		9	7	15.25	15.56	1.000	
	Rarely/never	27	58	84.38	80.56	0.786		49	36	83.05	80.00	0.799	
Sweets (biscuits, cakes, chocolate, etc.)	Two or more times a day	2	1	6.25	1.39	0.223	0.186	1	2	1.69	4.44	0.577	0.341
	Once a day	3	12	9.38	16.67	0.384		7	8	11.86	17.78	0.414	
	Up to five times a week	7	7	21.88	9.72	0.121		8	6	13.56	13.33	1.000	
	Once or twice a week	12	24	37.50	33.33	0.824		18	18	30.51	40.00	0.406	
	Rarely/never	8	28	25.00	38.89	0.188		25	11	42.37	24.44	0.064	
Salty snacks (crackers, pretzels, chips, flips, popcorn, etc.)	Two or more times a day	2	4	6.25	5.56	1.000	0.920	2	4	3.39	8.89	0.399	0.615
	Once a day	5	10	15.63	13.89	0.772		7	8	11.86	17.78	0.414	
	Up to five times a week	5	7	15.63	9.72	0.507		7	5	11.86	11.11	1.000	
	Once or twice a week	10	26	31.25	36.11	0.663		23	13	38.98	28.89	0.306	
	Rarely/never	10	25	31.25	34.72	0.824		20	15	33.90	33.33	1.000	
Nuts (walnuts, almonds, hazelnuts, etc.)	Two or more times a day	0	0	0.00	0.00	n/a	0.214	0	0	0.00	0.00	n/a	0.490
	Once a day	1	9	3.13	12.50	0.169		5	5	8.47	11.11	0.743	
	Up to five times a week	1	0	3.13	0.00	0.308		0	1	0.00	2.22	0.433	
	Once or twice a week	12	22	37.50	30.56	0.504		22	12	37.29	26.67	0.295	
	Rarely/never	18	41	56.25	56.94	1.000		32	27	54.24	60.00	0.690	
Seeds (chia, pumpkin, flax, sesame, etc.)	Two or more times a day	0	1	0.00	1.39	1.000	0.174	1	0	1.69	0.00	1.000	0.452
	Once a day	3	6	9.38	8.33	1.000		5	4	8.47	8.89	1.000	
	Up to five times a week	5	2	15.63	2.78	0.286		4	3	6.78	6.67	1.000	
	Once or twice a week	7	17	21.88	23.61	1.000		17	7	28.81	15.56	0.159	
	Rarely/never	17	46	53.13	63.89	0.696		32	31	54.24	68.89	0.158	
Carbonated drinks and syrups	Two or more times a day	2	4	6.25	5.56	1.000	0.550	4	2	6.78	4.44	0.696	0.961
	Once a day	0	3	0.00	4.17	0.550		2	1	3.39	2.22	1.000	
	Up to five times a week	0	3	0.00	4.17	0.550		2	1	3.39	2.22	1.000	
	Once or twice a week	2	6	6.25	8.33	1.000		5	3	8.47	6.67	1.000	
	Rarely/never	28	56	87.50	77.78	0.290		46	38	77.97	84.44	0.460	
Natural juices (squeezed from fruit or purchased without added sugar, etc.)	Two or more times a day	3	9	9.38	12.50	0.750	0.940	4	8	6.78	17.78	0.121	0.485
	Once a day	4	9	12.50	12.50	1.000		8	5	13.56	11.11	0.773	
	Up to five times a week	3	7	9.38	9.72	1.000		5	5	8.47	11.11	0.743	
	Once or twice a week	6	17	18.75	23.61	0.800		14	9	23.73	20.00	0.812	
	Rarely/never	16	30	50.00	41.67	0.520		28	18	47.46	40.00	0.551	
Tea	Two or more times a day	4	5	12.50	6.94	0.620	0.323	4	5	6.78	11.11	0.496	0.557
	Once a day	3	3	9.38	4.17	0.545		2	4	3.39	8.89	0.399	
	Up to five times a week	3	9	9.38	12.50	1.000		6	6	10.17	13.33	0.759	
	Once or twice a week	5	5	15.63	6.94	0.650		7	3	11.86	6.67	0.509	
	Rarely/never	17	50	53.13	69.44	0.566		40	27	67.80	60.00	0.418	
Water	Two or more times a day	28	54	87.50	75.00	0.739	0.164	50	32	84.75	71.11	0.145	0.006
	Once a day	0	6	0.00	8.33	0.455		5	1	8.47	2.22	0.231	
	Up to five times a week	0	3	0.00	4.17	1.000		2	1	3.39	2.22	1.000	
	Once or twice a week	2	1	6.25	1.39	0.400		1	2	1.69	4.44	0.577	
	Rarely/never	2	8	6.25	11.11	0.628		1	9	1.69	20.00	0.002	

F—female, M—male, U—urban, R—rural; statistical significance *p* < 0.05.

**Table 4:** Dietary habits of respondents in relation to education level of parents.

Question		Mother’s Level of Education						Father’s Level of Education					
How Often Does Your Child with Down Syndrome Consume	Frequency of Consumption	HS (n = 55)	UD (n = 49)	HS (%)	UD (%)	*p*	*p* Global	HS (n = 72)	UD (n = 32)	HS (%)	UD (%)	*p*	*p* Global
White/semi-white bread	Two or more times a day	12	8	21.82	16.33	0.619	0.105	13	7	18.06	21.88	0.788	0.482
	Once a day	17	6	30.91	12.24	0.032		18	5	25.00	15.63	0.321	
	Up to five times a week	3	6	5.45	12.24	0.301		8	1	11.11	3.13	0.269	
	Once or twice a week	6	9	10.91	18.37	0.403		9	6	12.50	18.75	0.546	
	Rarely/never	17	20	30.91	40.82	0.312		24	13	33.33	40.63	0.511	
Black/wholemeal bread	Two or more times a day	4	2	7.27	4.08	0.681	0.631	6	0	8.33	0.00	0.174	0.575
	Once a day	6	4	10.91	8.16	0.746		7	3	9.72	9.38	1.000	
	Up to five times a week	3	4	5.45	8.16	0.704		5	2	6.94	6.25	1.000	
	Once or twice a week	6	10	10.91	20.41	0.276		10	6	13.89	18.75	0.562	
	Rarely/never	36	29	65.45	59.18	0.548		44	21	61.11	65.63	0.827	
Whole grains such as barley, oats, etc., in the form of grains, groats, or flour	Two or more times a day	5	5	9.09	10.20	1.000	0.882	7	3	9.72	9.38	1.000	0.691
	Once a day	14	14	25.45	28.57	0.826		21	7	29.17	21.88	0.483	
	Up to five times a week	3	5	5.45	10.20	0.471		4	4	5.56	12.50	0.247	
	Once or twice a week	13	10	23.64	20.41	0.814		17	6	23.61	18.75	0.798	
	Rarely/never	20	15	36.36	30.61	0.678		23	12	31.94	37.50	0.655	
Potatoes	Two or more times a day	2	1	3.64	2.04	1.000	0.125	3	0	4.17	0.00	0.551	0.216
	Once a day	3	0	5.45	0.00	0.245		3	0	4.17	0.00	0.551	
	Up to five times a week	5	2	9.09	4.08	0.443		4	3	5.56	9.38	0.673	
	Once or twice a week	37	43	67.27	87.76	0.019		52	28	72.22	87.50	0.130	
	Rarely/never	8	3	14.55	6.12	0.210		10	1	13.89	3.13	0.166	
Pasta	Two or more times a day	1	1	1.82	2.04	1.000	0.467	2	0	2.78	0.00	1.000	0.561
	Once a day	2	0	3.64	0.00	0.497		2	0	2.78	0.00	1.000	
	Up to five times a week	1	2	1.82	4.08	0.600		1	2	1.39	6.25	0.223	
	Once or twice a week	42	33	76.36	67.35	0.382		52	23	72.22	71.88	1.000	
	Rarely/never	9	13	16.36	26.53	0.236		15	7	20.83	21.88	1.000	
Rice	Two or more times a day	2	1	3.64	2.04	1.000	0.261	3	0	4.17	0.00	0.551	0.519
	Once a day	0	3	0.00	6.12	0.101		2	1	2.78	3.13	1.000	
	Up to five times a week	0	0	0.00	0.00	n/a		0	0	0.00	0.00	n/a	
	Once or twice a week	39	36	70.91	73.47	0.829		49	26	68.06	81.25	0.236	
	Rarely/never	14	9	25.45	18.37	0.480		18	5	25.00	15.63	0.321	
Milk	Two or more times a day	12	4	21.82	8.16	0.062	0.063	16	0	22.22	0.00	0.002	0.009
	Once a day	14	8	25.45	16.33	0.337		16	6	22.22	18.75	0.798	
	Up to five times a week	4	11	7.27	22.45	0.048		8	7	11.11	21.88	0.224	
	Once or twice a week	7	6	12.73	12.24	1.000		10	3	13.89	9.38	0.750	
	Rarely/never	18	20	32.73	40.82	0.421		22	16	30.56	50.00	0.078	
Fermented dairy products (yogurt, cream, kefir, etc.)	Two or more times a day	8	2	14.55	4.08	0.098	0.067	8	2	11.11	6.25	0.720	0.947
	Once a day	16	9	29.09	18.37	0.253		18	7	25.00	21.88	0.808	
	Up to five times a week	13	11	23.64	22.45	1.000		16	8	22.22	25.00	0.803	
	Once or twice a week	7	16	12.73	32.65	0.018		15	8	20.83	25.00	0.620	
	Rarely/never	11	11	20.00	22.45	0.813		15	7	20.83	21.88	1.000	
Cheese	Two or more times a day	0	0	0.00	0.00	n/a	0.625	0	0	0.00	0.00	n/a	0.345
	Once a day	6	2	10.91	4.08	0.276		5	3	6.94	9.38	0.699	
	Up to five times a week	4	5	7.27	10.20	0.732		4	5	5.56	15.63	0.129	
	Once or twice a week	21	20	38.18	40.82	0.842		29	12	40.28	37.50	0.831	
	Rarely/never	24	22	43.64	44.90	1.000		34	12	47.22	37.50	0.398	
Fruit	Two or more times a day	14	15	25.45	30.61	0.663	0.587	20	9	27.78	28.13	1.000	0.625
	Once a day	21	19	38.18	38.78	1.000		30	10	41.67	31.25	0.385	
	Up to five times a week	10	8	18.18	16.33	1.000		11	7	15.28	21.88	0.413	
	Once or twice a week	8	3	14.55	6.12	0.210		7	4	9.72	12.50	0.734	
	Rarely/never	2	4	3.64	8.16	0.417		4	2	5.56	6.25	1.000	
Vegetables	Two or more times a day	6	11	10.91	22.45	0.183	0.031	11	6	15.28	18.75	0.78	0.207
	Once a day	21	20	38.18	40.82	0.842		28	13	38.89	40.63	1.000	
	Up to five times a week	12	15	21.82	30.61	0.373		16	11	22.22	34.38	0.362	
	Once or twice a week	9	2	16.36	4.08	0.056		9	2	12.50	6.25	0.503	
	Rarely/never	7	1	12.73	2.04	0.063		8	0	11.11	0.00	0.102	
Meat	Two or more times a day	4	4	7.27	8.16	1.000	0.025	4	4	5.56	12.50	0.267	0.119
	Once a day	13	14	23.64	28.57	0.656		19	8	26.39	25.00	1.000	
	Up to five times a week	16	25	29.09	51.02	0.028		25	16	34.72	50.00	0.433	
	Once or twice a week	16	5	29.09	10.20	0.026		17	4	23.61	12.50	0.428	
	Rarely/never	6	1	10.91	2.04	0.117		7	0	9.72	0.00	0.190	
Meat products (salami, hot dogs, pâté, etc.)	Two or more times a day	2	2	3.64	4.08	1.000	0.008	1	3	1.39	9.38	0.099	0.046
	Once a day	6	3	10.91	6.12	0.495		9	0	12.50	0.00	0.059	
	Up to five times a week	11	1	20.00	2.04	0.005		10	2	13.89	6.25	0.505	
	Once or twice a week	16	11	29.09	22.45	0.506		17	10	23.61	31.25	0.644	
	Rarely/never	20	32	36.36	65.31	0.006		35	17	48.61	53.13	0.856	
Eggs	Two or more times a day	1	1	1.82	2.04	1.000	0.298	2	0	2.78	0.00	1.000	0.417
	Once a day	3	2	5.45	4.08	1.000		2	3	2.78	9.38	0.325	
	Up to five times a week	2	7	3.64	14.29	0.080		5	4	6.94	12.50	0.463	
	Once or twice a week	27	25	49.09	51.02	1.000		38	14	52.78	43.75	0.711	
	Rarely/never	22	14	40.00	28.57	0.302		25	11	34.72	34.38	1.000	
Fish	Two or more times a day	0	0	0.00	0.00	n/a	0.359	0	0	0.00	0.00	n/a	0.172
	Once a day	0	0	0.00	0.00	n/a		0	0	0.00	0.00	n/a	
	Up to five times a week	1	2	1.82	4.08	0.628		1	2	1.39	6.25	0.236	
	Once or twice a week	26	29	47.27	59.18	0.529		36	19	50.00	59.38	0.721	
	Rarely/never	28	18	50.91	36.73	0.372		35	11	48.61	34.38	0.439	
Olive oil	Two or more times a day	3	4	5.45	8.16	0.704	0.003	3	4	4.17	12.50	0.211	0.039
	Once a day	9	9	16.36	18.37	0.801		11	7	15.28	21.88	0.586	
	Up to five times a week	3	13	5.45	26.53	0.005		9	7	12.50	21.88	0.390	
	Once or twice a week	6	9	10.91	18.37	0.403		9	6	12.50	18.75	0.556	
	Rarely/never	34	14	61.82	28.57	0.0008		40	8	55.56	25.00	0.076	
Coconut oil	Two or more times a day	0	0	0.00	0.00	n/a	0.210	0	0	0.00	0.00	n/a	0.014
	Once a day	0	1	0.00	2.04	0.471		0	1	0.00	3.13	0.314	
	Up to five times a week	0	2	0.00	4.08	0.22		0	2	0.00	6.25	0.101	
	Once or twice a week	3	5	5.45	10.20	0.471		4	4	5.56	12.50	0.267	
	Rarely/never	52	41	94.55	83.67	0.109		68	25	94.44	78.13	0.637	
Refined oils and saturated fats (sunflower oil, canola oil, lard, etc.)	Two or more times a day	1	1	1.82	2.04	1.000	0.623	1	1	1.39	3.13	0.528	0.736
	Once a day	6	9	10.91	18.37	0.403		9	6	12.50	18.75	0.556	
	Up to five times a week	12	6	21.82	12.24	0.299		13	5	18.06	15.63	1.000	
	Once or twice a week	15	12	27.27	24.49	0.825		18	9	25.00	28.13	0.818	
	Rarely/never	21	21	38.18	42.86	0.691		31	11	43.06	34.38	0.69	
Butter	Two or more times a day	0	0	0.00	0.00	n/a	0.507	0	0	0.00	0.00	n/a	0.051
	Once a day	3	3	5.45	6.12	1.000		3	3	4.17	9.38	0.38	
	Up to five times a week	3	7	5.45	14.29	0.185		4	6	5.56	18.75	0.081	
	Once or twice a week	16	13	29.09	26.53	0.829		19	10	26.39	31.25	0.822	
	Rarely/never	33	26	60.00	53.06	0.554		46	13	63.89	40.63	0.276	
Fast food (burgers, pizza, etc.)	Two or more times a day	1	0	1.82	0.00	1.000	0.492	1	0	1.39	0.00	1.000	0.923
	Once a day	1	0	1.82	0.00	1.000		1	0	1.39	0.00	1.000	
	Up to five times a week	1	0	1.82	0.00	1.000		1	0	1.39	0.00	1.000	
	Once or twice a week	10	6	18.18	12.24	0.43		12	4	16.67	12.50	0.774	
	Rarely/never	42	43	76.36	87.76	0.203		57	28	79.17	87.50	0.756	
Sweets (biscuits, cakes, chocolate, etc.)	Two or more times a day	3	0	5.45	0.00	0.245	0.159	2	1	2.78	3.13	1.000	0.304
	Once a day	10	5	18.18	10.20	0.278		11	4	15.28	12.50	1.000	
	Up to five times a week	8	6	14.55	12.24	0.781		8	6	11.11	18.75	0.353	
	Once or twice a week	20	16	36.36	32.65	0.837		29	7	40.28	21.88	0.078	
	Rarely/never	14	22	25.45	44.90	0.042		22	14	30.56	43.75	0.264	
Salty snacks (crackers, pretzels, chips, flips, popcorn, etc.)	Two or more times a day	6	0	10.91	0.00	0.028	0.040	5	1	6.94	3.13	0.664	0.318
	Once a day	11	4	20.00	8.16	0.101		11	4	15.28	12.50	1.000	
	Up to five times a week	6	6	10.91	12.24	1.000		11	1	15.28	3.13	0.099	
	Once or twice a week	16	20	29.09	40.82	0.223		24	12	33.33	37.50	0.824	
	Rarely/never	16	19	29.09	38.78	0.306		21	14	29.17	43.75	0.179	
Nuts (walnuts, almonds, hazelnuts, etc.)	Two or more times a day	0	0	0.00	0.00	n/a	0.090	0	0	0.00	0.00	n/a	0.375
	Once a day	4	6	7.27	12.24	0.511		5	5	6.94	15.63	0.277	
	Up to five times a week	0	1	0.00	2.04	0.471		1	0	1.39	0.00	1.000	
	Once or twice a week	14	20	25.45	40.82	0.142		23	11	31.94	34.38	0.824	
	Rarely/never	37	22	67.27	44.90	0.029		43	16	59.72	50.00	0.396	
Seeds (chia, pumpkin, flax, sesame, etc.)	Two or more times a day	0	1	0.00	2.04	0.471	0.562	0	1	0.00	3.13	0.308	0.465
	Once a day	4	5	7.27	10.20	0.732		8	1	11.11	3.13	0.269	
	Up to five times a week	3	4	5.45	8.16	0.704		5	2	6.94	6.25	1.000	
	Once or twice a week	11	13	20.00	26.53	0.489		16	8	22.22	25.00	0.803	
	Rarely/never	37	26	67.27	53.06	0.162		43	20	59.72	62.50	0.831	
Carbonated drinks and syrups	Two or more times a day	4	2	7.27	4.08	0.681	0.713	4	2	5.56	6.25	1.000	0.981
	Once a day	1	2	1.82	4.08	0.300		2	1	2.78	3.13	1.000	
	Up to five times a week	1	2	1.82	4.08	0.600		2	1	2.78	3.13	1.000	
	Once or twice a week	3	5	5.45	10.20	0.471		5	3	6.94	9.38	0.699	
	Rarely/never	46	38	83.64	77.55	0.464		59	25	81.94	78.13	0.788	
Natural juices (squeezed from fruit or purchased without added sugar, etc.)	Two or more times a day	8	4	14.55	8.16	0.369	0.223	7	5	9.72	15.63	0.507	0.837
	Once a day	7	6	12.73	12.24	1.000		9	4	12.50	12.50	1.000	
	Up to five times a week	8	2	14.55	4.08	0.098		7	3	9.72	9.38	1.000	
	Once or twice a week	9	14	16.36	28.57	0.160		15	8	20.83	25.00	0.62	
	Rarely/never	23	23	41.82	46.94	0.693		34	12	47.22	37.50	0.398	
Tea	Two or more times a day	7	2	12.73	4.08	0.167	0.448	8	1	11.11	3.13	0.269	0.700
	Once a day	4	2	7.27	4.08	0.681		4	2	5.56	6.25	1.000	
	Up to five times a week	7	5	12.73	10.20	0.765		9	3	12.50	9.38	0.751	
	Once or twice a week	4	6	7.27	12.24	0.511		6	4	8.33	12.50	0.493	
	Rarely/never	33	34	60.00	69.39	0.412		45	22	62.50	68.75	0.658	
Water	Two or more times a day	39	43	70.91	87.76	0.053	0.259	55	27	76.39	84.38	0.442	0.261
	Once a day	4	2	7.27	4.08	0.681		4	2	5.56	6.25	1.000	
	Up to five times a week	2	1	3.64	2.04	1.000		1	2	1.39	6.25	0.223	
	Once or twice a week	3	0	5.45	0.00	0.245		3	0	4.17	0.00	0.551	
	Rarely/never	7	3	12.73	6.12	0.328		9	1	12.50	3.13	0.169	

HS—completed high school, UD—completed university degree (bachelor’s; master’s; PhD), statistical significance *p* < 0.05.

**Table 5:** Dietary habits of respondents in relation to mother’s employment status and share of food costs in family income.

Question		Mother’s Employment Status						Share of Food Costs in Family Income					
How Often Does Your Child with Down Syndrome Consume	Frequency of Consumption	UE (n = 39)	E (n = 65)	UE (%)	E (%)	*p*	*p* Global	>50% (n = 35)	<50% (n = 69)	>50% (%)	<50% (%)	*p*	*p* Global
White/semi-white bread	Two or more times a day	13	7	33.33	10.77	0.009	0.005	11	9	31.43	13.04	0.035	0.255
	Once a day	10	13	25.64	20.00	0.626		8	15	22.86	21.74	1.000	
	Up to five times a week	2	7	5.13	10.77	0.478		2	7	5.71	10.14	0.714	
	Once or twice a week	1	14	2.56	21.54	0.008		4	11	11.43	15.94	0.769	
	Rarely/never	13	24	33.33	36.92	0.833		10	27	28.57	39.13	0.386	
Black/wholemeal bread	Two or more times a day	3	3	7.69	4.62	0.669	0.602	3	3	8.57	4.35	0.402	0.192
	Once a day	3	7	7.69	10.77	0.740		2	8	5.71	11.59	0.489	
	Up to five times a week	1	6	2.56	9.23	0.252		2	5	5.71	7.25	1.000	
	Once or twice a week	5	11	12.82	16.92	0.780		2	14	5.71	20.29	0.082	
	Rarely/never	27	38	69.23	58.46	0.302		26	39	74.29	56.52	0.090	
Whole grains such as barley, oats, etc., in the form of grains, groats, or flour	Two or more times a day	4	6	10.26	9.23	1.000	0.646	3	7	8.57	10.14	1.000	0.155
	Once a day	12	16	30.77	24.62	0.503		11	17	31.43	24.64	0.489	
	Up to five times a week	1	7	2.56	10.77	0.253		0	8	0.00	11.59	0.049	
	Once or twice a week	8	15	20.51	23.08	0.812		6	17	17.14	24.64	0.460	
	Rarely/never	14	21	35.90	32.31	0.831		15	20	42.86	28.99	0.190	
Potatoes	Two or more times a day	3	0	7.69	0.00	0.050	0.019	1	2	2.86	2.90	1.000	0.479
	Once a day	2	1	5.13	1.54	0.555		2	1	5.71	1.45	0.261	
	Up to five times a week	4	3	10.26	4.62	0.421		3	4	8.57	5.80	0.685	
	Once or twice a week	24	56	61.54	86.15	0.007		24	56	68.57	81.16	0.217	
	Rarely/never	6	5	15.38	7.69	0.323		5	6	14.29	8.70	0.501	
Pasta	Two or more times a day	2	0	5.13	0.00	0.138	0.093	0	2	0.00	2.90	0.549	0.330
	Once a day	1	1	2.56	1.54	1.000		2	0	5.71	0.00	0.111	
	Up to five times a week	0	3	0.00	4.62	0.290		1	2	2.86	2.90	1.000	
	Once or twice a week	25	50	64.10	76.92	0.180		24	51	68.57	73.91	0.645	
	Rarely/never	11	11	28.21	16.92	0.217		8	14	22.86	20.29	0.802	
Rice	Two or more times a day	2	1	5.13	1.54	0.554	0.085	2	1	5.71	1.45	0.261	0.064
	Once a day	3	0	7.69	0.00	0.050		2	1	5.71	1.45	0.261	
	Up to five times a week	0	0	0.00	0.00	n/a		0	0	0.00	0.00	n/a	
	Once or twice a week	26	49	66.67	75.38	0.372		20	55	57.14	79.71	0.021	
	Rarely/never	8	15	20.51	23.08	0.812		11	12	31.43	17.39	0.134	
Milk	Two or more times a day	7	9	17.95	13.85	0.586	0.972	7	9	20.00	13.04	0.395	0.709
	Once a day	8	14	20.51	21.54	1.000		9	13	25.71	18.84	0.452	
	Up to five times a week	6	9	15.38	13.85	1.000		4	11	11.43	15.94	0.769	
	Once or twice a week	5	8	12.82	12.31	1.000		3	10	8.57	14.49	0.536	
	Rarely/never	13	25	33.33	38.46	0.676		12	26	34.29	37.68	0.831	
Fermented dairy products (yogurt, cream, kefir, etc.)	Two or more times a day	8	2	20.51	3.08	0.006	0.042	3	7	8.57	10.14	1.000	0.255
	Once a day	6	19	15.38	29.23	0.155		10	15	28.57	21.74	0.473	
	Up to five times a week	9	15	23.08	23.08	1.000		6	18	17.14	26.09	0.338	
	Once or twice a week	7	16	17.95	24.62	0.474		5	18	14.29	26.09	0.216	
	Rarely/never	9	13	23.08	20.00	0.805		11	11	31.43	15.94	0.08	
Cheese	Two or more times a day	0	0	0.00	0.00	n/a	0.366	0	0	0.00	0.00	n/a	0.106
	Once a day	4	4	10.26	6.15	0.469		5	3	14.29	4.35	0.115	
	Up to five times a week	1	8	2.56	12.31	0.148		3	6	8.57	8.70	1.000	
	Once or twice a week	16	25	41.03	38.46	0.838		9	32	25.71	46.38	0.056	
	Rarely/never	18	28	46.15	43.08	0.839		18	28	51.43	40.58	0.305	
Fruit	Two or more times a day	13	16	33.33	24.62	0.372	0.633	10	19	28.57	27.54	1.000	0.060
	Once a day	12	28	30.77	43.08	0.298		8	32	22.86	46.38	0.032	
	Up to five times a week	6	12	15.38	18.46	0.793		7	11	20.00	15.94	0.596	
	Once or twice a week	5	6	12.82	9.23	0.743		6	5	17.14	7.25	0.176	
	Rarely/never	3	3	7.69	4.62	0.669		4	2	11.43	2.90	0.176	
Vegetables	Two or more times a day	6	11	15.38	16.92	1.000	0.104	5	12	14.29	17.39	0.785	0.308
	Once a day	12	29	30.77	44.62	0.214		14	27	40.00	39.13	1.000	
	Up to five times a week	9	18	23.08	27.69	0.651		6	21	17.14	30.43	0.164	
	Once or twice a week	8	3	20.51	4.62	0.018		6	5	17.14	7.25	0.176	
	Rarely/never	4	4	10.26	6.15	0.469		4	4	11.43	5.80	0.438	
Meat	Two or more times a day	5	3	12.82	4.62	0.148	0.116	3	5	8.57	7.25	1.000	0.002
	Once a day	10	17	25.64	26.15	1.000		14	13	40.00	18.84	0.032	
	Up to five times a week	11	30	28.21	46.15	0.097		5	36	14.29	52.17	0.002	
	Once or twice a week	8	13	20.51	20.00	1.000		8	13	22.86	18.84	0.616	
	Rarely/never	5	2	12.82	3.08	0.100		5	2	14.29	2.90	0.041	
Meat products (salami, hot dogs, pâté, etc.)	Two or more times a day	3	1	7.69	1.54	0.147	0.233	2	2	5.71	2.90	0.601	0.244
	Once a day	3	6	7.69	9.23	1.000		5	4	14.29	5.80	0.16	
	Up to five times a week	6	6	15.38	9.23	0.359		6	6	17.14	8.70	0.212	
	Once or twice a week	12	15	30.77	23.08	0.489		8	19	22.86	27.54	0.645	
	Rarely/never	15	37	38.46	56.92	0.105		14	38	40.00	55.07	0.213	
Eggs	Two or more times a day	2	0	5.13	0.00	0.138	0.020	2	0	5.71	0.00	0.111	0.020
	Once a day	4	1	10.26	1.54	0.064		3	2	8.57	2.90	0.332	
	Up to five times a week	4	5	10.26	7.69	0.725		0	9	0.00	13.04	0.027	
	Once or twice a week	13	39	33.33	60.00	0.015		16	36	45.71	52.17	0.678	
	Rarely/never	16	20	41.03	30.77	0.297		14	22	40.00	31.88	0.513	
Fish	Two or more times a day	0	0	0.00	0.00	n/a	0.129	0	0	0.00	0.00	n/a	0.202
	Once a day	0	0	0.00	0.00	n/a		0	0	0.00	0.00	n/a	
	Up to five times a week	1	2	2.56	3.08	1.000		0	3	0.00	4.35	0.549	
	Once or twice a week	16	39	41.03	60.00	0.070		16	39	45.71	56.52	0.308	
	Rarely/never	22	24	56.41	36.92	0.067		19	27	54.29	39.13	0.151	
Olive oil	Two or more times a day	0	7	0.00	10.77	0.043	0.120	3	4	8.57	5.80	0.685	0.858
	Once a day	9	9	23.08	13.85	0.286		6	12	17.14	17.39	1.000	
	Up to five times a week	4	12	10.26	18.46	0.400		4	12	11.43	17.39	0.569	
	Once or twice a week	7	8	17.95	12.31	0.565		4	11	11.43	15.94	0.769	
	Rarely/never	19	29	48.72	44.62	0.691		18	30	51.43	43.48	0.533	
Coconut oil	Two or more times a day	0	0	0.00	0.00	n/a	0.142	0	0	0.00	0.00	n/a	0.555
	Once a day	1	0	2.56	0.00	0.375		0	1	0.00	1.45	1.000	
	Up to five times a week	0	2	0.00	3.08	0.527		1	1	2.86	1.45	1.000	
	Once or twice a week	1	7	2.56	10.77	0.253		1	7	2.86	10.14	0.262	
	Rarely/never	37	56	94.87	86.15	0.202		33	60	94.29	86.96	0.327	
Refined oils and saturated fats (sunflower oil, canola oil, lard, etc.)	Two or more times a day	1	1	2.56	1.54	1.000	0.970	1	1	2.86	1.45	1.000	0.823
	Once a day	6	9	15.38	13.85	1.000		6	9	17.14	13.04	0.568	
	Up to five times a week	6	12	15.38	18.46	0.793		6	12	17.14	17.39	1.000	
	Once or twice a week	11	16	28.21	24.62	0.818		7	20	20.00	28.99	0.356	
	Rarely/never	15	27	38.46	41.54	0.838		15	27	42.86	39.13	0.833	
Butter	Two or more times a day	0	0	0.00	0.00	n/a	0.017	0	0	0.00	0.00	n/a	0.089
	Once a day	3	3	7.69	4.62	0.669		4	2	11.43	2.90	0.176	
	Up to five times a week	0	10	0.00	15.38	0.012		2	8	5.71	11.59	0.489	
	Once or twice a week	15	14	38.46	21.54	0.073		6	23	17.14	33.33	0.106	
	Rarely/never	21	38	53.85	58.46	0.686		23	36	65.71	52.17	0.214	
Fast food (burgers, pizza, etc.)	Two or more times a day	1	0	2.56	0.00	0.375	0.420	1	0	2.86	0.00	0.337	0.464
	Once a day	1	0	2.56	0.00	0.375		0	1	0.00	1.45	1.000	
	Up to five times a week	0	1	0.00	1.54	1.000		0	1	0.00	1.45	1.000	
	Once or twice a week	5	11	12.82	16.92	0.780		7	9	20.00	13.04	0.395	
	Rarely/never	32	53	82.05	81.54	1.000		27	58	77.14	84.06	0.427	
Sweets (biscuits, cakes, chocolate, etc.)	Two or more times a day	3	0	7.69	0.00	0.050	0.198	1	2	2.86	2.90	1.000	0.951
	Once a day	6	9	15.38	13.85	1.000		6	9	17.14	13.04	0.568	
	Up to five times a week	4	10	10.26	15.38	0.561		4	10	11.43	14.49	0.769	
	Once or twice a week	15	21	38.46	32.31	0.531		11	25	31.43	36.23	0.669	
	Rarely/never	11	25	28.21	38.46	0.395		13	23	37.14	33.33	0.828	
Salty snacks (crackers, pretzels, chips, flips, popcorn, etc.)	Two or more times a day	6	0	15.38	0.00	0.002	0.016	2	4	5.71	5.80	1.000	0.077
	Once a day	7	8	17.95	12.31	0.565		9	6	25.71	8.70	0.035	
	Up to five times a week	4	8	10.26	12.31	1.000		1	11	2.86	15.94	0.056	
	Once or twice a week	10	26	25.64	40.00	0.201		11	25	31.43	36.23	0.669	
	Rarely/never	12	23	30.77	35.38	0.673		12	23	34.29	33.33	1.000	
Nuts (walnuts, almonds, hazelnuts, etc.)	Two or more times a day	0	0	0.00	0.00	n/a	0.014	0	0	0.00	0.00	n/a	0.001
	Once a day	0	10	0.00	15.38	0.012		1	9	2.86	13.04	0.159	
	Up to five times a week	1	0	2.56	0.00	0.375		1	0	2.86	0.00	0.337	
	Once or twice a week	9	25	23.08	38.46	0.132		5	29	14.29	42.03	0.004	
	Rarely/never	29	30	74.36	46.15	0.008		28	31	80.00	44.93	0.0008	
Seeds (chia, pumpkin, flax, sesame, etc.)	Two or more times a day	0	1	0.00	1.54	1.000	0.204	0	1	0.00	1.45	1.000	0.524
	Once a day	5	4	12.82	6.15	0.290		3	6	8.57	8.70	1.000	
	Up to five times a week	1	6	2.56	9.23	0.251		1	6	2.86	8.70	0.419	
	Once or twice a week	6	18	15.38	27.69	0.229		6	18	17.14	26.09	0.338	
	Rarely/never	27	36	69.23	55.38	0.214		25	38	71.43	55.07	0.138	
Carbonated drinks and syrups	Two or more times a day	3	3	7.69	4.62	0.669	0.234	5	1	14.29	1.45	0.016	0.078
	Once a day	0	3	0.00	4.62	0.290		2	1	5.71	1.45	0.261	
	Up to five times a week	2	1	5.13	1.54	0.555		1	2	2.86	2.90	1.000	
	Once or twice a week	5	3	12.82	4.62	0.148		2	6	5.71	8.70	0.714	
	Rarely/never	29	55	74.36	84.62	0.210		25	59	71.43	85.51	0.114	
Natural juices (squeezed from fruit, or purchased without added sugar, etc.)	Two or more times a day	4	8	10.26	12.31	1.000	0.664	4	8	11.43	11.59	1.000	0.650
	Once a day	5	8	12.82	12.31	1.000		4	9	11.43	13.04	1.000	
	Up to five times a week	6	4	15.38	6.15	0.170		2	8	5.71	11.59	0.489	
	Once or twice a week	8	15	20.51	23.08	0.812		6	17	17.14	24.64	0.460	
	Rarely/never	16	30	41.03	46.15	0.685		19	27	54.29	39.13	0.151	
Tea	Two or more times a day	5	4	12.82	6.15	0.290	0.273	3	6	8.57	8.70	1.000	0.503
	Once a day	3	3	7.69	4.62	0.669		3	3	8.57	4.35	0.402	
	Up to five times a week	4	8	10.26	12.31	1.000		4	8	11.43	11.59	1.000	
	Once or twice a week	1	9	2.56	13.85	0.086		1	9	2.86	13.04	0.159	
	Rarely/never	26	41	66.67	63.08	0.833		24	43	68.57	62.32	0.665	
Water	Two or more times a day	28	54	71.79	83.08	0.217	0.387	26	56	74.29	81.16	0.452	0.058
	Once a day	2	4	5.13	6.15	1.000		1	5	2.86	7.25	0.661	
	Up to five times a week	1	2	2.56	3.08	1.000		0	3	0.00	4.35	0.549	
	Once or twice a week	2	1	5.13	1.54	0.555		3	0	8.57	0.00	0.036	
	Rarely/never	6	4	15.38	6.15	0.17		5	5	14.29	7.25	0.298	

UE—unemployed, E—employed, statistical significance *p* < 0.05.

## Data Availability

The original contributions presented in this study are included in this article; further inquiries can be directed to the corresponding author.
